# An Information Theoretic Condition for Perfect Reconstruction

**DOI:** 10.3390/e26010086

**Published:** 2024-01-19

**Authors:** Idris Delsol, Olivier Rioul, Julien Béguinot, Victor Rabiet, Antoine Souloumiac

**Affiliations:** 1 Laboratoire de Traitement et Communication de l’Information, Télécom Paris, Institut Polytechnique de Paris, 91120 Palaiseau, France; idris.delsol@telecom-paris.fr (I.D.); julien.beguinot@telecom-paris.fr (J.B.); or victor.rabiet@ens.fr (V.R.); 2 Département de Mathématiques et Applications, École Nationale Supérieure, 75005 Paris, France; 3 CEA-List, Université Paris-Saclay, 91120 Palaiseau, France; antoine.souloumiac@cea.fr

**Keywords:** information lattice, common information, complementary information, Rajski distance, Shannon distance, dependency coefficient, relative redundancy, convex envelope, perfect reconstruction

## Abstract

A new information theoretic condition is presented for reconstructing a discrete random variable *X* based on the knowledge of a set of discrete functions of *X*. The reconstruction condition is derived from Shannon’s 1953 lattice theory with two entropic metrics of Shannon and Rajski. Because such a theoretical material is relatively unknown and appears quite dispersed in different references, we first provide a synthetic description (with complete proofs) of its concepts, such as total, common, and complementary information. The definitions and properties of the two entropic metrics are also fully detailed and shown to be compatible with the lattice structure. A new geometric interpretation of such a lattice structure is then investigated, which leads to a necessary (and sometimes sufficient) condition for reconstructing the discrete random variable *X* given a set $\left\{X_{1},\ldots ,X_{n}\right\}$ of elements in the lattice generated by *X*. Intuitively, the components $X_{1},\ldots ,X_{n}$ of the original source of information *X* should not be globally “*too far away*” from *X* in the entropic distance in order that *X* is reconstructable. In other words, these components should not overall have too *low of a dependence* on *X*; otherwise, reconstruction is impossible. These geometric considerations constitute a starting point for a possible novel “perfect reconstruction theory”, which needs to be further investigated and improved along these lines. Finally, this condition is illustrated in five specific examples of perfect reconstruction problems: the reconstruction of a symmetric random variable from the knowledge of its sign and absolute value, the reconstruction of a word from a set of linear combinations, the reconstruction of an integer from its prime signature (fundamental theorem of arithmetic) and from its remainders modulo a set of coprime integers (Chinese remainder theorem), and the reconstruction of the sorting permutation of a list from a minimal set of pairwise comparisons.

A movement is accomplished in six stagesAnd the seventh brings return.The seven is the number of the young lightIt forms when darkness is increased by one.Change returns successGoing and coming without error.Action brings good fortune.Sunset, sunrise.
*Syd Barrett, Chapter 24 (Pink Floyd).*


## 1. Introduction

We consider the problem of perfectly reconstructing a discrete random variable *X*, based on the knowledge of a finite set $X_{1}$, $X_{2}$, …, $X_{n}$ of deterministic processings or transformations of *X*, denoted $f_{i}$, such that $X_{i}=f_{i}\left(X\right)$. Intuitively, the components $X_{i}$ are assumed to carry only a partial amount of the “information” present in *X*, and the perfect reconstruction of *X* would only be possible if the combination of the “information” in $X_{1}$, $X_{2}$, …, $X_{n}$ is enough to contain all the original “information” in *X*. Such intuitive considerations expressed in the language of information are very common in signal processing and in many other scientific fields; but, they were never mathematically formalized as far as the authors know. This article aims at formalizing precisely this trivial and vague intuition. Such a task implies, in particular, an accurate definition of “information”.

The Shannon’s 1948 classical information theory [1] cannot really answer this question as it is rather a theory of the measure of information rather than of the information itself. Fortunately, a “true information” theory was also developed by Claude Shannon in a relatively unknown 1953 article [2], which is *not* what is generally referred to as “Shannon’s information theory”. Said briefly, the information is defined there as an equivalence class of discrete random variables. A partial order on a set of classes allows one to build a lattice structure called the *information lattice*, which is made metric by the introduction of two related entropic distances.

“*Claude [Shannon] did not like the term* ‘*information theory*’” recalls Robert Fano, a colleague of Shannon’s working at MIT, who died almost a century old just seven years ago. In one of his last interviews [3], he said, “*You see, the term ’information theory’ suggests that it’s a theory about information, but it’s not. It’s about the transmission of information, not the information. Many people just didn’t understand that”*. Fano is of course referring to Shannon’s famous theory in his 1948 seminal paper [1], which he entitled, “ a mathematical theory of *communication*  ”—not information. But, very early on, it was the term “information” that prevailed. The entropy H(X) of a discrete random variable *X* is presented as the measure of “*information* contained in *X*”, and the notion of the *mutual information* I(X;Y) between two variables *X* and *Y*, introduced precisely by the same Robert Fano in his course at MIT [4], quickly became central to the teaching of the theory. Moreover, the very first historical article on the theory, barely three years after its birth, is entitled “*A history of the theory of information*” [5].

This sudden craze for “information” in the early 1950s eventually became somewhat of a bore for Shannon, who in 1956, in his famous editorial, *The Bandwagon*  [6] warned against the excesses of such popularity: “*It will be all too easy for our somewhat artificial prosperity to collapse overnight when we realize that the use of a few exciting words like information, entropy, redundancy, does not solve all our problems*”.

Under these conditions, it is understandable that Shannon wanted to go further: If several, unrelated, random variables can have the same *quantity* of information *H*, how can information itself be defined? Shannon presented a very brief summary of his findings (without proofs) at the International Congress of Mathematicians (ICM) in 1950 [7] and in a small, relatively unknown article [2] published in 1953 in the very first issue of what was to become the *IEEE Transactions on Information Theory*.

The remainder of this article is organized as follows. Section 2 presents in detail the Shannon theory of the lattice of information with complete proofs, and Section 3 does the same for the two entropic distances proposed, respectively, by Shannon and Rajski. The corresponding geometric point of view is further developed in Section 4. Two conditions of perfect reconstruction, a necessary one and a sufficient one, are then derived in Section 5. Finally, the condition is applied to five specific examples in Section 6.

Section 2, Section 3, and Section 4.1 are a deepening of the article [8] previously published (in French) by four of the authors.

## 2. What Is Information? A Detailed Study of Shannon’s Information Lattice

For simplicity, we consider with Shannon discrete random variables *X*, which take a *finite* number of values in some alphabet X. This amounts to considering all the random variables X:Ω→X defined on a given probability space (Ω,P(Ω),P), where the underlying universe Ω is finite and P(Ω) is the power set of Ω.

### 2.1. Definition of the “True” Information

Quite arguably, the *information* contained in a discrete source or random variable *X* should not be confused with the “measure of quantity of information” such as the entropy H(X). Shannon’s idea [2] is that this information contained in *X* should in fact be defined as *X* itself.Of course, any reversible encoding of *X* must be regarded as the *same* information, since one moves from one representation to another without loss of information. This amounts, in modern language, to the following definition:

**Definition 1**  (“True” information)**.** *The *information* (contained in) X is the equivalence class of X for the equivalence relation:*

(1) X≡Y⟺Y=f(X)andX=g(Y)a.s.(almostsurely)

*for two deterministic functions f and g.*

**Proof.**  Relation ≡ is evidently reflexive (take *f* and *g* to be the identity function) and symmetric (by permuting the roles of *f* and *g* in the definition). It is also transitive by composition: if X≡Y and Y≡Z, there exists *f*, *g*, *h*, and *k* such that Y=f(X), X=g(Y), and Y=h(Z), Z=k(Y) a.s.; then, X=g(h(Z))=g∘h(Z) and Z=k∘f(X) a.s.  □

**Proposition 1.**  *X≡Y if and only if (iff) there exists a bijective function h such that Y=h(X) a.s.*

**Proof.**  If X≡Y, then there exist two deterministic functions *f* and *g* such that X=f(Y) and Y=g(X) a.s. Thus, X=f(g(X)) a.s. Then, for every value X=x with non-zero probability, f∘g(x)=x. Hence, f∘g coincides with the identity function a.s. Since the problem is symmetric in *X* and *Y*, g∘f also coincides with the identity function a.s. Thus, h=g is bijective from the set of values that *X* can take with non-zero probability to the set of values that *Y* can take with non-zero probability, and we have Y=g(X)=h(X) a.s.

Conversely, if Y=h(X) a.s. with bijective *h*, then $X=h^{-1}\left(Y\right)$ a.s.; hence, X≡Y.  □

As suggested by Rajski [9], the equivalence between *X* and *Y* can be characterized by way of their joint probability matrix:

**Proposition 2**  (Matrix characterization)**.** *If we restrain *Ω* to the elements of the non-zero probability measure, X≡Y iff the matrix of joint probabilities P(X=x,Y=y) is a permutation matrix.*

**Proof.**  By Proposition 1, X≡Y iff there exists a bijective function *h* such that Y=h(X) a.s. Thus, to each outcome of *X* corresponds exactly one outcome of *Y* and vice versa, which is equivalent to saying that the matrix of joint probabilities is a permutation matrix. □

In the following, we shall denote (without possible confusion) *X* the equivalence class of the variable *X*, and thus, X=Y, the equality between the two classes *X* and *Y* (rather than X≡Y).

With this definition, it is clear that the equivalence relation is compatible with any functional relation Y=f(X). If *f* is not bijective, it is tempting to say that there is *less* information in *Y* than in *X*, hence the following partial order.

**Definition 2**  (Partial order)**.**

(2) X≥Y⟺Y=f(X)a.s.

*for some deterministic function f.*

We also write Y≤X. We are not necessarily considering real-valued variables, so the order X≥Y has nothing to do with the order in R.

**Proposition 3.**  *The relation ≥ is indeed a partial order on the set of equivalence classes of the relation ≡ defined above.*

**Proof.**  We first show that the relation ≡ is compatible with the relation ≥. Let $X_{1}$, $X_{2}$, and $Y_{1}$, $Y_{2}$ be such that $X_{1}\equiv X_{2}$ and $Y_{1}\equiv Y_{2}$. Then, if $X_{1}\geq Y_{1}$, there exists a deterministic function *f* such that $Y_{1}=f\left(X_{1}\right)$ a.s. Since $X_{1}\equiv X_{2}$, there exists a bijective *h* such that $X_{1}=h\left(X_{2}\right)$ a.s.; hence, $Y_{1}=f\circ h\left(X_{2}\right)$ a.s. and $X_{2}\geq Y_{1}$. Likewise, since $Y_{1}\equiv Y_{2}$, there exists a bijective *g* such that $Y_{2}=g\left(Y_{1}\right)$ a.s., so $Y_{2}=g\circ f\circ h\left(X_{2}\right)$ a.s.; hence, $X_{2}\geq Y_{2}$. This shows that the relation ≥ is well defined on the set of equivalence classes of the relation ≡.

We now show that ≥ is indeed a partial order:

- *Reflexivity*: X=Id(X) so X≥X.
- *Antisymmetry*: If X≥Y and Y≥X, X=f(Y) a.s., and Y=g(X) a.s. for deterministic functions *f* and *g*, so X≡Y.
- *Transitivity*: If X≥Y and Y≥Z, then there exist two deterministic functions *f* and *g* such that: Z=g(Y) a.s. and Y=f(X) a.s. Then, Z=g(f(X)) a.s.; hence, X≥Z. □

### 2.2. Structure of the Information Lattice: Joint Information; Common Information

Beyond the partial order, Shannon [2] established the natural mathematical structure of information: it is a *lattice*, i.e., two variables X,Y always admit a maximum X∨Y and a minimum X∧Y. Let us recall that these quantities (necessarily unique if they exist) are defined by the relations:

(3) $$\begin{array}{c} (X\leq Z\;\mathrm{and}\;Y\leq Z)⟺X\vee Y\leq Z,\\ (X\geq Z\;\mathrm{and}\;Y\geq Z)⟺X\wedge Y\geq Z. \end{array}$$

Shannon, in his paper [2], used Boolean notations instead, X+Y for X∨Y and X·Y for X∧Y.

**Proposition 4**  (Joint information)**.** *The joint information X∨Y of X and Y is the random pair X∨Y=(X,Y).*

**Proof.**  If *X* and *Y* are functions of *Z*, then the pair (X,Y) is also a function of *Z*. Conversely, since *X* and *Y* are functions of (X,Y), if (X,Y) is a function of *Z*, then so are *X* and *Y*.  □

The definition of X∧Y (*common information*) is more difficult and was not made explicit by Shannon. Following Gács and Körner [10], let us adopt the following definition:

**Definition 3.**  *We say that x∈X and y∈Y* communicate*, denoted by x∼y, if there exists a path $xy_{1}x_{1}y_{2}\cdots y_{n}x_{n}y$ in which all transitions are of non-zero probability: $P(X=x,Y=y_{1})>0$, $P(Y=y_{1},X=x_{1})>0$, …, $P(X=x_{n},Y=y)>0$.*

For convenience, we also write y∼x when *x* and *y* communicate. Strictly speaking, the relation x∼y is not an equivalence relation because *x* and *y* do not belong to the same set. However, it has similar properties:

**Proposition 5.**  *The relation ∼ on the set of pairs (x,y) for which P(X=x)>0 and P(Y=y)>0 is transitive in the sense that $x_{1}∼y_{1}$, $y_{1}∼x_{2}$, and $x_{2}∼y_{2}$ implies $x_{1}∼y_{2}$.*

**Proof.**  If $x_{1}∼y_{1}$, $y_{1}∼x_{2}$, and $x_{2}∼y_{2}$, then there exists a path from $x_{1}$ to $y_{1}$, another from $y_{1}$ to $x_{2}$, and a leastone from $x_{2}$ to $y_{2}$, whose transitions are of non-zero probability. The concatenated path from $x_{1}$ to $y_{2}$ has non-zero transition probabilities; hence, $x_{1}∼y_{2}$.  □

**Definition 4**  (Communication class)**.** *If x∼y, we define the communication class C(x,y) as the set of all $\left(x^{\prime },y^{\prime }\right)$ such that $x^{\prime }∼y$ and $x∼y^{\prime }$.*

Thus, by transitivity, $C\left(x,y\right)=C\left(x^{\prime },y^{\prime }\right)$ for all $\left(x^{\prime },y^{\prime }\right)$ in the communication class, so that two classes are either equal or distinct. Therefore, the distinct communication classes partition the set of all values (x,y) for which P(X=x)>0 and P(Y=y)>0. We may identify any communication class *C* with its characteristic function $1_{(x,y)\in C}$ so that C(X,Y) is a binary random variable.

**Proposition 6**  (Common information)**.** *The common information X∧Y of X and Y is X∧Y=C(X,Y).*

**Proof.**  If Z=f(X)=g(Y) a.s., then *Z* is constant for each pair (x,y) such that x∼y; in other words, *Z* is a function of the class C(X,Y).  □

**Remark 1.**  *In order to compute the common information between X and Y in practice, one has to fully determine the communication classes, which is only possible if there is a finite number of classes, each of which contains a finite number of elements. In other words, X and Y should take a finite number of values. This is the reason why we restrict ourselves to finitely valued variables in this paper.*

**Remark 2.**  *As in any lattice, X≤Y is equivalent to saying that X∨Y=Y or that X∧Y=X.*

### 2.3. Computing Common Information

As shown in the previous section, the definition of common information is not a simple one, but one can compute it efficiently using the following algorithm. Given two variables *X* and *Y*, this algorithm turns the joint probability matrix of (X,Y) into a *block-diagonal* matrix, where each block corresponds to each communication class.

Let *X* and *Y* be two random variables taking values in X and Y, respectively. Consider the graph G=(V,E) whose vertices *V* are X∪Y and such that the vertices *x* and *y* of *V* are connected by an edge if and only if P(X=x,Y=y)>0. Hence, *G* is fully described by the joint probability matrix $P_{X,Y}$. Furthermore, this is a bipartite graph (no edge connects two vertices $x_{1}$ and $x_{2}$ belonging to X or two vertices $y_{1}$ and $y_{2}$ belonging to Y).

Then, the communication classes C(X,Y) correspond to the *connected components* of *G*. Indeed, a connected component *C* is a subset of *V* such that each of its elements is accessible to all the others by a path in the subgraph (C,E). So, for any two vertices *x*, *y* in the connected component *C*, there exists $y_{1}$, $x_{1}$, …, $y_{k}$, $x_{k}$ such that all the edges $\left(x,y_{1}\right)$, $\left(y_{1},x_{1}\right)$,…, $\left(y_{k},x_{k}\right)$, $\left(x_{k},y\right)$ belong to *E*, that is all the transition probabilities between these vertices are non-zero, which is equivalent to saying that they belong to the same communication class. Now, it is known that the connected components of *G* can be determined by a depth-first search.

We propose an algorithm, whose pseudo-code is given in Algorithm 1, that takes as the input the joint probability matrix $P_{X,Y}$ and outputs a block-diagonal form of $P_{X,Y}$ representing the common information X∧Y, an array storing the permutation of the columns of $P_{X,Y}$, and an array storing the permutation of the rows $P_{X,Y}$. Since the matrix $P_{X,Y}$ is sufficient to fully describe *G*, we adapt the depth-first search algorithm to browse the rows and columns of the matrix $P_{X,Y}$ to find which of its rows and columns must be swapped in order to write this matrix in a block-diagonal form. In this algorithm (Algorithm 1), the *i*th row of $P_{X,Y}$ will be represented by the pair (r,i) and the *j*th column by the pair (c,j).    
**Algorithm 1:** Algorithm to compute the common information.1:input $P_{X,Y}$: $n_{R}\times n_{C}$ matrix                       ▹ Joint probability matrix2:$\sigma_{R}\leftarrow$ array of integers of length $n_{R}$                  ▹ Rows’ permutation vector3:$\sigma_{C}\leftarrow$ array of integers of length $n_{C}$               ▹ Columns’ permutation vector4:S← empty stack             ▹ Stack contains row indices (r,i) or column indices (c,j)5:push (r,0) into stack *S*                        ▹ First row put into stack6:bottom←1                      ▹ Bottommost row index not yet assigned7:$\mathrm{up}\leftarrow n_{R}-1$              ▹ Uppermost row index that may have non-zero entries8:left←0                       ▹ Leftmost column index not yet assigned9:$\mathrm{right}\leftarrow n_{C}-1$           ▹ Rightmost column index that may have non-zero entries10:**while** There is an unmarked row or column **do**11:    **while** *S* is not empty **do**12:        (s,i)←S.pop()    ▹ The pop() operation removes the top stack element and returns it.13:        **if** (s,i) is not marked **then**14:           mark (s,i)15:           **if** s=r **then**                        ▹ Current index *i* is a row index16:               **for** left≤j≤right **do**                       ▹ Scan all columns17:                   **if** $P_{X,Y}\left(i,j\right)>0$ **then**18:                       push (c,j) into stack *S*19:                       $\sigma_{C}\left[j\right]\leftarrow \mathrm{left}$; swap columns left and *j* in $P_{X,Y}$20:                       left←left+121:                   **end if**22:               **end for**23:               **if** all entries on *i*th row are zeros **then**24:                   $\sigma_{R}\left[i\right]\leftarrow \mathrm{up}$; swap rows *i* and up in $P_{X,Y}$25:                   up←up−126:               **end if**27:           **else**                       ▹ Current index *i* is a column index28:               **for** bottom≤j≤up **do**                      ▹ Scan all rows29:                   **if** $P_{X,Y}\left(j,i\right)>0$ **then**30:                       push (r,j) into stack *S*31:                       $\sigma_{R}\left[j\right]\leftarrow \mathrm{bottom}$; swap rows bottom and *j* in $P_{X,Y}$32:                       bottom←bottom+133:                   **end if**34:               **end for**35:               **if** all entries on *i*th column are zeros **then**36:                   $\sigma_{C}\left[i\right]\leftarrow \mathrm{right}$; swap columns *i* and right in $P_{X,Y}$37:                   right←right−138:               **end if**39:           **end if**40:        **end if**41:    **end while**                                 ▹ Empty Stack42:    **if** there is an unmarked *i*th row (r,i) **then**43:        push (r,i) into stack *S*44:    **else if** there is an unmarked *j*th column (c,j) **then**45:        push (c,j) into stack *S*46:    **end if**47:**end while**                          ▹ All rows and columns marked48:**return**$P_{X,Y}$, $\sigma_{R}$, $\sigma_{C}$

The complexity of this algorithm can be determined as follows. Let n=Card(X)+Card(Y) be the sum of the alphabet sizes on which *X* and *Y* take their values, i.e., the sum of the number of rows of $P_{X,Y}$ and the number of columns of $P_{X,Y}$. The algorithm passes through each row and column at most once. Indeed, for the index of a row or column to enter the stack, it must be *unmarked*, but as soon as we put it on the stack, we mark it. Then, each time the index of a row or a column is unstacked, we look at each coefficient of the corresponding row or column. Therefore, our algorithm looks at each coefficient of the joint probability matrix $P_{X,Y}$ exactly once. Four elementary operations are performed each time we cross a non-zero coefficient. Thus, the algorithm’s complexity is quadratic in *n*.

Notice that the output of our algorithm gives a visualization of the common information: The stochastic matrix P(X=x,Y=y) is written, after the permutation of the rows/columns, in the “block-diagonal” form

(4) $$P_{X,Y}=\left(\begin{array}{ccccccc} C_{1}\\ \; & C_{2} & \; & 0\\ \; & \; & \ddots \\ 0 & \; & & C_{k}\\ \; & \; & & \; & 0\\ \; & \; & & \; & & \ddots \\ \; & \; & & \; & & \; & 0 \end{array}\right)$$

where *k*, the number of blocks, is maximal. The *k* rectangular matrices then represent the *k* different equivalence classes, the probability P(C(X,Y)=i) being the sum of all entries in block $C_{i}$.

### 2.4. Boundedness and Complementedness: Null, Total, and Complementary Information

**Proposition 7**  (Null information; total information)**.** *The information lattice is* bounded*, i.e., it admits a minimum of 0 and a maximum of 1, such that, for any X, 0≤X≤1:*

- *The minimal element 0 (“null information”) is the equivalence class of all* deterministic *variables. Thus, X=0 means that X is a deterministic variable.*
- *The maximal element 1 (“total information”) of the lattice is the equivalence class of the identity function Id on Ω.*

**Proof.**  If *X* is any random variable and Z=c a.s. is any deterministic variable, then it is clear that Z=f(X), where *f* is the constant function *c*. Letting 0 be the equivalence class of constant variables, one has 0≤X for all *X*.

Also, for any random variable *X*, X=X∘Id; hence, X≤Id. Letting 1 be the equivalence class of the identity function on Ω, one has X≤1 for all *X*.  □

**Proposition 8**  (Complementary information)**.** *The information lattice is* complemented*, i.e., any X≤Y admits a complement Z (“complementary information”) such that X∨Z=Y and X∧Z=0.*

This *Z* is the information missing from *X* to obtain *Y*: It allows *Y* to be reconstructed from *X* without requiring more information than necessary. Shannon in [2] did not say how to determine it. The following proof gives an explicit construction:

**Proof.**  Since X≤Y, we simply have X=X∧Y=C(X,Y). Thus, a given class C(X,Y)=x has only one value X=x per class, corresponding in general to several values of *Y*, say $y_{1}^{x},y_{2}^{x},\ldots ,y_{k_{x}}^{x}$. Now, let $Z\in \{1,\ldots ,k_{X}\}$ be the unique index such that $Y=Y_{Z}^{X}$.

By construction, Z≤X∨Y=Y, and since X≤Y, one also has X∨Z≤Y. But, the formula $Y=Y_{Z}^{X}$ shows that Y≤X∨Z; hence, the equality X∨Z=Y holds.

Finally, the value Z=1 connects each pair (x,z), so there is only one class according to (X,Z), i.e., X∧Z=0.  □

**Figure 1:**
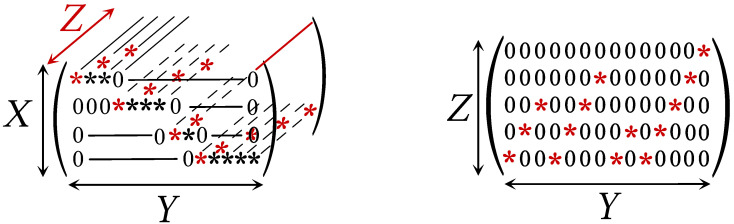
Construction of the complementary information *Z* allowing passing from *X* to *Y*. The stochastic tensor of (X,Y,Z) representing $P_{X,Y,Z}$ has non-zero entries marked in red. The distribution $P_{Z}$ of *Z* is obtained by marginalizing the tensor on the *Z*-axis.

This construction can be visualized on the stochastic tensor of (X,Y,Z) described in Figure 1.

**Remark 3.**  *The complementary information Z is* not *uniquely determined by X and Y. In the above construction, it depends on how the values of Y are indexed by the class X=x.*

### 2.5. Computing the Complementary Information

Given X≤Y, Algorithm 2 determines a random variable *Z* corresponding to the complementary information from *X* to *Y*. This algorithm takes as the input the joint probability matrix $P_{X,Y}$ in its block-diagonal form and outputs the tensor of the joint probability $P_{X,Y,Z}$, where X∨Z=Y and X∧Z=0. The tensor is built by spreading the non-zero coefficients of the joint probability matrix $P_{X,Y}$ on the *Z*-axis as shown in Figure 1.
**Algorithm 2:** Algorithm for computing the complementary information.1:input $P_{X,Y}$: $n_{R}\times n_{C}$ matrix           ▹ Joint probability matrix2:k←0                           ▹ Z index3:**for** 0≤i<nR **do**4:    **for** 0≤j<nC **do**5:        **if** $P_{X,Y}\left(i,j\right)>0$ **then**6:           $P_{X,Y,Z}\left(i,j,k\right)\leftarrow P_{X,Y}\left(i,j\right)$7:           k←k+18:        **end if**9:    **end for**10:    k←011:**end for**12:**return** $P_{X,Y,Z}$

The algorithm looks at each coefficient of the joint probability matrix $P_{X,Y}$ exactly once and performs at most two elementary operations for each coefficient it processes. Therefore, it is quadratic in n=Card(X)+Card(Y) (since the number of coefficients in the matrix $P_{X,Y}$ is quadratic in *n*).

### 2.6. Relationship between Complementary Information and Functional Representation

There is a striking resemblance between Proposition 8 and the “functional representation lemma”, which has been used in recent years in various applications of information theory for network coding (see Appendix B, pp. 626–627, of [11]).

For the convenience of the notations, we write X⊥Y if X∧Y=0 (null common information) and write X⫫Y iff *X* and *Y* are *independent*. It is easily seen (see Remark 4 below) that X⫫Y⇒X⊥Y. Now, Proposition 8 and the “functional representation lemma” can be rewritten as follows.

**Lemma 1**  (Complementary information lemma (Proposition 8))**.** 

(5) ∀X≤Y,∃Z⊥Xs.t.Y=X∨Z.

**Lemma 2**  (Functional representation lemma ([11]))**.** 

(6) ∀X,Y,∃Z⫫Xs.t.Y≤X∨Z.

Thus, compared to the “complementary information lemma”, the “functional representation lemma” (i) has a general assumption of *X* and *Y* (*X* need not be a function of *Y*), but (ii) requires a stronger condition on *Z* (Z⫫X instead of Z⊥X) and (iii) has a weaker conclusion (*Y* is only a function of *X* and *Z*). It would be interesting to further investigate the relationship between these two lemmas since it is apparent that one lemma cannot be deduced from the other.

### 2.7. Is the Information Lattice a Boolean Algebra?

Interestingly, it was Shannon who, as early as 1938 in his master’s thesis, used the *Boolean algebra* to study relay-based circuits—“the most important master’s thesis of the century” for which Shannon received the Alfred Noble prize (not to be confused with the Alfred Nobel Prize) in 1940. But, alas, as Shannon noted, his information lattice is *not* a Boolean algebra. It would have been one if it were *distributive* (∧ distributive with respect to ∨ or vice versa), since a Boolean algebra is, by definition, a distributive complemented bounded lattice. However:

**Proposition 9.**  *The information lattice is not distributive.*

**Indirect proof**.  In any Boolean algebra, the complement is unique. As seen above, this is not the case for the information lattice. □

**Direct proof.**  As a direct second proof, we provide an explicit counterexample to distributivity. Consider the probability space (Ω,P(Ω),P), where Ω={0,1,2,3} and P is the uniform probability measure, and define X(ω)=0 if ω is even, X(ω)=1 otherwise. Now, let $Z_{1}$, $Z_{2}$ be given as in Table 1 below. As we read in the table, $\left(X\wedge Z_{1}\right)\vee \left(X\wedge Z_{2}\right)=0$ is constant, while $X\wedge (Z_{1}\vee Z_{2})$ is not. Therefore, $\left(X\wedge Z_{1}\right)\vee \left(X\wedge Z_{2}\right)\neq X\wedge \left(Z_{1}\vee Z_{2}\right)$, and the information lattice is not distributive. □

## 3. Metric Properties of the Information Lattice

### 3.1. Information and Information Measures

First of all, it is immediate to check the compatibility of the information lattice with respect to the entropy or the mutual information as logarithmic measures of information.

We use the following standard notations. The entropy of *X* is denoted H(X). If *X* takes values in X of size *N*, then the entropy of *X* satisfies H(X)≤logN with equality iff *X* is uniformly distributed. Here, “log” refers to the logarithm taken to *any* base. The conditional entropy of *X* given *Y* is denoted H(X|Y), and the mutual information between *X* and *Y* is denoted I(X;Y).

**Proposition 10.**  *Entropy, conditional entropy, and mutual information are compatible with the definition of information as an equivalence class:*

**Proof.** 

- *Entropy*: If X≡Y, there exist functions *f* and *g* such that Y=f(X) a.s. (hence, H(Y)≤H(X)) and X=g(Y) a.s. (hence, H(X)≤H(Y)). Thus, H(X)=H(Y).
- *Conditional entropy*: Let $X_{1}\equiv X_{2}$ with *f* and *g* be two functions such that $X_{1}=f\left(X_{2}\right)$ and $X_{2}=g\left(X_{1}\right)$ a.s. Then, $H\left(X_{1}|Y\right)=H\left(f\left(X_{2}\right)|Y\right)\leq H\left(X_{2}|Y\right)$. Similarly, $H\left(X_{2}|Y\right)=H\left(g\left(X_{1}\right)|Y\right)\leq H\left(X_{1}|Y\right)$. Therefore, $H\left(X_{1}|Y\right)=H\left(X_{2}|Y\right)$. Finally, if $Y_{1}\equiv Y_{2}$ with two functions *h* and *k* such that $Y_{1}=h\left(Y_{2}\right)$ and $Y_{2}=k\left(Y_{1}\right)$ a.s., then $H\left(X|Y_{1}\right)=H\left(X|h\left(Y_{2}\right)\right)\geq H\left(X|Y_{2},h\left(Y_{2}\right)\right)=H\left(X|Y_{2}\right)$ and likewise $H\left(X|Y_{2}\right)=H\left(X|k\left(Y_{1}\right)\right)\geq H\left(X|Y_{1},k\left(Y_{1}\right)\right)=H\left(X|Y_{1}\right)$. Therefore, $H\left(X|Y_{1}\right)=H\left(X|Y_{2}\right)$.
- *Mutual information*: Since I(X;Y)=H(X)−H(X|Y), compatibility follows from the two previous cases. □

We then have some obvious connections:

**Proposition 11**  (Partial order and conditional entropy)**.**

(7) X≤Y⟺H(X|Y)=0

*In particular, H is “order-preserving” (greater information implies higher entropy):*

(8) X≤Y⟹H(X)≤H(Y).

*Also, X≤Y with H(X)=H(Y) implies X=Y.*

*Finally, H(X)≥0 for all X, with equality H(X)=0 iff X=0.*

**Proof.**  H(X|Y)=0 means that H(X|Y=y)=0 for all y∈Y, which amounts to saying that *X* is deterministic equal to f(y) given Y=y. In other words, X=f(Y) a.s. We then have H(X)=H(X)−H(X|Y)=I(X;Y)=H(Y)−H(Y|X)≤H(Y).

Next, suppose that X≤Y and H(X)=H(Y). By the chain rule, H(X,Y)=H(X)+H(Y|X)=H(Y)+H(X|Y). Therefore, it follows from the equality H(X)=H(Y) that H(Y|X)=H(X|Y). But, since X≤Y, H(X|Y)=0; hence, H(Y|X)=0 also, that is Y≤X. This shows equivalence Y=X.

Finally, since X≥0 for all *X*, H(X)≥H(0)=0, and it is well known that the entropy H(X) is zero if and only if the variable *X* is deterministic, that is X=0. □

**Figure 2:**
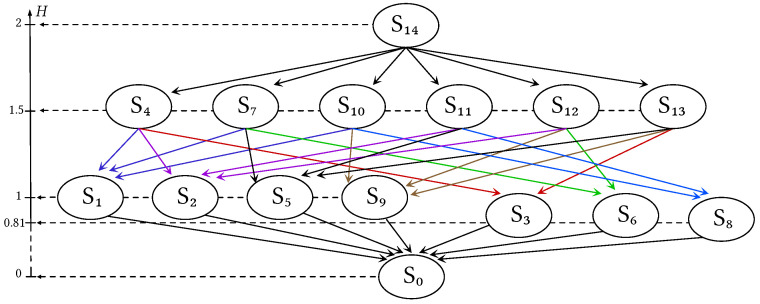
Hasse diagram of the information lattice defined on a universe Ω of size 4 with uniform probability. There are 15 different random variables corresponding to the 15 different ways to partition Ω. The corresponding entropies are, in descending order: 2; 1.5; 1; ≈0.81, and 0 bits.

An example of an information lattice with associated entropies is given in Figure 2.

### 3.2. Common Information vs. Mutual Information

**Proposition 12.**  *The entropy of the joint information is the joint entropy, i.e., H(X∨Y)=H(X,Y).*

**Proof.**  Obvious, since X∨Y=(X,Y). □

**Figure 3:**
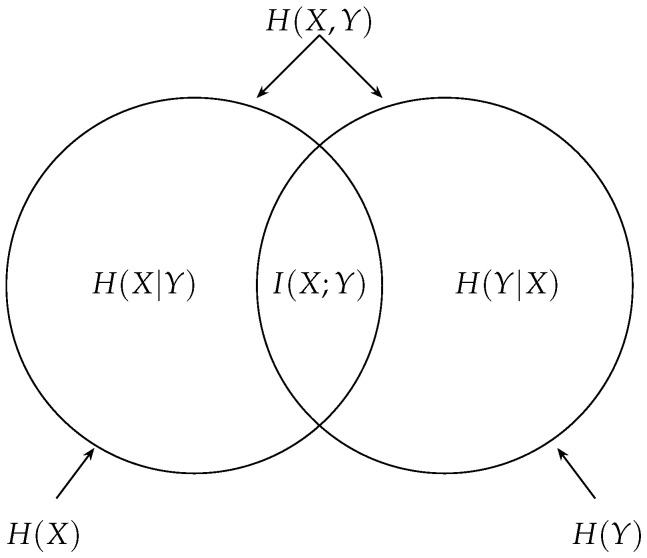
Usual Venn diagram in information theory.

One may wonder by analogy with the usual Venn diagram in information theory (Figure 3) if the entropy of joint information is equal to the mutual information: Is it true that H(X∧Y)=I(X;Y)? The answer is *no*, as shown next. Proposition 13 is implicit in [10] and made explicit by Wyner in [12], who credits a private communication from Kaplan.

**Proposition 13.**  *H(X∧Y)≤I(X;Y) always, with equality H(X∧Y)=I(X;Y) iff one can write X=(U,W) and Y=(V,W), where U and V are conditionally independent given W.*

**Proof.**  Let W=X∧Y. Since W≤X and W≤Y, by complementarity, we can write X=W∨U=(U,W) and Y=W∨V=(V,W). By the chain rule for mutual information, I(X;Y)=I(U,W;V,W)=I(W;V,W)+I(U;V,W|W)=H(W)+I(U;V|W)≥H(W) with equality iff *U* and *V* are conditionally independent given *W*. □

**Remark 4.**  *In particular, if X and Y are independent, they have null common information X∧Y=0. However, common information H(X∧Y) can be far less [10] than mutual information I(X;Y).*

**Remark 5.**  *Notice that the case of equality corresponds to the case where the matrix blocks $C_{i}$ in (4) are stochastic matrices of two independent variables X,Y knowing W=i, i.e., matrices of rank one.*

**Remark 6.**  *Shannon’s notion of common information should not be confused with the well-known Wyner’s accepting of “common information”, which is defined as the maximum of I(X,Y;W) when X and Y are conditionally independent knowing W. This quantity is not less, but* greater *than the mutual information I(X;Y) [12].*

### 3.3. Submodularity of Entropy on the Information Lattice

From the results in [13], we can show that entropy is *submodular* on the information lattice:

**Proposition 14**  (Submodularity of entropy)**.** *H(X∨Y)+H(X∧Y)≤H(X)+H(Y).*

**Proof.**  Since X∧Y≤Y, H(Y)=H(Y,X∧Y)=H(X∧Y)+H(Y|X∧Y). But, since X∧Y≤X, H(Y|X∧Y)≥H(Y|X∧Y,X)=H(Y|X)=H(X∨Y)−H(X). Combining gives the announced inequality. □

**Remark 7.**  *Submodularity is in fact equivalent to the inequality H(X∧Y)≤I(X;Y) of Proposition 13, since H(X)+H(Y)−H(X∨Y)=H(X)+H(Y)−H(X,Y)=I(X;Y).*

**Remark 8.**  *The submodularity property of entropy that is generally studied in the information theory literature is with respect to the set lattice (or algebra), where the entropy is that of a collection of random variables indexed by some index set (thus considered as a set function). Such considerations have been greatly developed in recent years; see, e.g., [14]. By contrast, it is the information lattice that is considered here. It can be easily shown using Proposition 13 that the two notions of submodularity coincide for collections of independent random variables.*

### 3.4. Two Entropic Metrics: Shannon Distance; Rajski Distance

Since X=Y⟺(X≤YandX≥Y), according to Proposition 11, it suffices that H(X|Y)+H(Y|X)=0 in order for *X* and *Y* to be equivalent: X=Y. Shannon [2] noted that this defines a *distance*, which makes the information lattice a *metric* space:

**Proposition 15**  (Shannon’s entropic distance)**.** *D(X,Y)=H(X|Y)+H(Y|X) is a distance over the information lattice:*

**Proof.** 

- *Positivity*: As just noted above, D(X,Y)≥0 vanishes only when X=Y.
- *Symmetry*: D(X,Y)=D(Y,X) is obvious by the commutativity of addition.
- *Triangular inequality*: First note that H(X|Z)≤H(X,Y|Z)=H(X|Y,Z)+H(Y|Z)≤H(X|Y)+H(Y|Z). By permuting *X* and *Z*, we also obtain that H(Z|X)≤H(Z|Y)+H(Y|X). Summing up the two inequalities, we obtain the triangular inequality D(X,Z)=H(X|Z)+H(Z|X)≤H(X|Y)+H(Y|X)+H(Y|Z)+H(Z|Y)=D(X,Y)+D(Y,Z). □

It is interesting to note that this is not the only distance (nor the only topology). By normalizing D(X,Y) by the joint entropy H(X,Y), we obtain another distance metric:

**Proposition 16**  (Rajski’s entropic distance [9])**.** *$d\left(X,Y\right)=\frac{D(X,Y)}{H(X,Y)}$ (with the convention d(0,0)=0) is a distance taking values in [0,1].*

Notice that normalization by H(X,Y) is valid when *X* and *Y* are non-deterministic since X≠0 and Y≠0 implies H(X,Y)>0.

**Proof.**  First of all, symmetry d(X,Y)=d(Y,X) is obvious, and positivity follows from that of *D*. We follow Horibe [15] to prove the triangular inequality. One may always assume non-deterministic random variables. Observe that:

(9) $$\frac{H(X|Y)}{H(X,Y)}=\frac{H(X|Y)}{H(X|Y)+H(Y)}\geq \frac{H(X|Y)}{H(X|Y)+H(Y,Z)}=\frac{H(X|Y)}{H(X|Y)+H(Y|Z)+H(Z)}$$

and

(10) $$\frac{H(Y|Z)}{H(Y,Z)}=\frac{H(Y|Z)}{H(Y|Z)+H(Z)}\geq \frac{H(Y|Z)}{H(X|Y)+H(Y|Z)+H(Z)}.$$

Summing (9) and (10) yields

(11) $$\frac{H(X|Y)}{H(X,Y)}+\frac{H(Y|Z)}{H(Y,Z)}\geq \frac{H(X|Y)+H(Y|Z)}{H(X|Y)+H(Y|Z)+H(Z)}.$$

Now, from the above proof of the triangular inequality of *D*, one has H(X|Y)+H(Y|Z)≥H(X|Z). Noting that a≥b>0 and c≥0 imply $\frac{a}{a+c}\geq \frac{b}{b+c}$, we obtain

(12) $$\frac{H(X|Y)+H(Y|Z)}{H(X|Y)+H(Y|Z)+H(Z)}\geq \frac{H(X|Z)}{H(X|Z)+H(Z)}=\frac{H(X|Z)}{H(X,Z)}.$$

Therefore,

(13) $$\frac{H(X|Y)}{H(X,Y)}+\frac{H(Y|Z)}{H(Y,Z)}\geq \frac{H(X|Z)}{H(X,Z)}.$$

Permuting the roles of *X* and *Z* gives

(14) $$\frac{H(Y|X)}{H(X,Y)}+\frac{H(Z|Y)}{H(Y,Z)}\geq \frac{H(Z|X)}{H(X,Z)}.$$

Summing (13) and (14), we conclude that d(X,Y)+d(Y,Z)≥d(X,Z). □

**Remark 9.**  *Rajski’s distance between two variables X and Y can be visualized as the* Jaccard distance *between the region corresponding to X and the region corresponding to Y in the Venn diagram of Figure 3. The Jaccard (or Jaccard–Tanimoto) distance [16] between two sets A and B is defined by $d_{J}\left(A,B\right)=\frac{\left|A\Delta B\right|}{\left|A\cup B\right|}$, where *Δ* is the symmetric difference between A and B. Thus, if A and B are, respectively, the regions corresponding to X and to Y in the Venn diagram, we have: H(X,Y)=|A∪B|, H(X|Y)=|A∖B| and H(Y|X)=|B∖A|. Thus, $\frac{H(X|Y)+H(Y|X)}{H(X,Y)}=\frac{\left|(A\setminus B)\cup (B\setminus A)\right|}{A\cup B}=\frac{\left|A\Delta B\right|}{\left|A\cup B\right|}$.*

### 3.5. Dependency Coefficient

From the Rajski distance, we can define a quantity that measures the *dependence* between two non-deterministic (i.e., non-zero) random variables *X* and *Y*.

**Definition 5**  (Dependency coefficient)**.** *For all non-zero elements X and Y of the information lattice, their* dependency coefficient *is ρ(X,Y)=1−d(X,Y)∈[0,1].*

**Proposition 17.**  *The dependency coefficient can be seen as normalized mutual information: $\rho \left(X,Y\right)=\frac{I(X;Y)}{H(X,Y)}$.*

**Proof.**  One has $1-d\left(X,Y\right)=1-\frac{H(X|Y)+H(Y|X)}{H(X,Y)}=\frac{H(X,Y)-H(X|Y)-H(Y|X)}{H(X,Y)}$, where the numerator =H(X)+H(Y|X)−H(X|Y)−H(Y|X)=I(X;Y). □

**Proposition 18.**  *For non-deterministic X and Y, one has 0≤ρ(X,Y)≤1, where ρ(X,Y)=0 vanishes (equivalently, d(X,Y)=1) iff X and Y are independent and ρ(X,Y)=1 (equivalently, d(X,Y)=0) iff X=Y are equivalent.*

**Proof.**  If *X* and *Y* are independent, then I(X;Y)=0; hence, ρ(X,Y)=0. If *X* and *Y* are equivalent, then d(X,Y)=0 and ρ(X,Y)=1−d(X,Y)=1. Since 0≤I(X;Y)≤H(X)≤H(X,Y), $0\leq \rho \left(X,Y\right)=\frac{I(X;Y)}{H(X,Y)}\leq 1$. □

**Remark 10.**  *The property of ρ in Proposition 18 is similar to the usual property of the linear correlation coefficient. However, while two independent random variables have zero correlation (but not conversely), the corresponding converse property holds for the dependence coefficient since two random variables are independent if and only if ρ(X,Y)=0.*

### 3.6. Discontinuity and Continuity Properties

Perhaps the biggest flaw in Shannon’s lattice information theory [2] is that the different constructions of elements in the lattice (e.g., common and complementary information) do not actually depend on the *values* of the probabilities involved, but only on whether they are equal to or different from zero. Thus, a small perturbation on the probabilities can greatly influence the results. As an illustration, we have the following.

**Proposition 19**  (Discontinuity of common information)**.** *The application (X,Y)↦X∧Y is* discontinuous *in the metric lattice with distance D (or d).*

**Proof.**  Let $\left(X_{\varepsilon },Y_{\varepsilon }\right)$ be defined by the stochastic matrix:

(15) $$P_{X,Y}=\left(\begin{array}{ccccc} \frac{1-\varepsilon }{N} & \frac{\varepsilon }{N} & 0 & \cdots & 0\\ 0 & \frac{1-\varepsilon }{N} & \frac{\varepsilon }{N} & \cdots & 0\\ \vdots & \vdots & \vdots & \ddots & \ddots \\ \frac{\varepsilon }{N} & 0 & \cdots & 0 & \frac{1-\varepsilon }{N} \end{array}\right).$$

Since there is a single class of communication, common information $X_{\varepsilon }\wedge Y_{\varepsilon }=0$ is zero for every ε>0. By contrast, when ε=0, $X_{\varepsilon }\wedge Y_{\varepsilon }$ is uniformly distributed among *N* communication classes. Consequently, $D(X_{\varepsilon }\wedge Y_{\varepsilon },0)=0$ for any ε>0, whereas $D\left(X_{0}\wedge Y_{0},0\right)=H\left(X_{0}\wedge Y_{0}\right)=\log N$ is arbitrarily large for ε=0. □

However, it should be noted that the joint information ∨ is continuous with respect to Shannon’s distance. In fact, we have the following.

**Proposition 20.**  *For any X, $X^{\prime }$, Y, and $Y^{\prime }$,*

(16) $$D\left(X\vee Y,X^{\prime }\vee Y^{\prime }\right)\leq D\left(X,X^{\prime }\right)+D\left(Y,Y^{\prime }\right).$$

**Proof.**  One has

(17) $$\begin{array}{cc} H(X\vee Y|X^{\prime }\vee Y^{\prime }) & =H(X,Y|X^{\prime },Y^{\prime })\\ \; & \overset{\left(a\right)}{=}H\left(X|X^{\prime },Y^{\prime }\right)+H\left(Y|X^{\prime },Y^{\prime },X\right)\\ \; & \overset{\left(b\right)}{\leq }H\left(X|X^{\prime }\right)+H\left(Y|Y^{\prime }\right). \end{array}$$

where (a) is the consequence of the chain rule and (b) is due to the fact that conditioning reduces entropy. Since *X*, $X^{\prime }$ and *Y*, $Y^{\prime }$ play a symmetrical role in (b), we can permute the roles of *X*, $X^{\prime }$ and *Y*, $Y^{\prime }$, which gives $H\left(X^{\prime }\vee Y^{\prime }|X\vee Y\right)\leq H\left(X^{\prime }|X\right)+H\left(Y^{\prime }|Y\right)$. Summing both inequalities yields the result. □

**Remark 11.**  *In particular, for $X=X^{\prime }$, for any X,Y,Z,*

(18) D(X∨Y,X∨Z)≤D(Y,Z).

*In other words, joining the same X can only reduce the Shannon distance: in this respect, the joining operator Y↦X∨Y is a contraction operator.*

Furthermore, the entropy, the conditional entropy, and the mutual information are continuous with respect to the entropic distance of Shannon. Indeed, we have the following inequalities (see Problem 3.5 in [17]):

**Proposition 21.**  *For all X, Y, $X^{\prime }$, and $Y^{\prime }$:*

*(i)*  *|H(X)−H(Y)|≤D(X,Y).*
*(ii)*  *$|H\left(X,Y\right)-H\left(X^{\prime },Y^{\prime }\right)|\leq D\left(X,X^{\prime }\right)+D\left(Y,Y^{\prime }\right)$.*
*(iii)*  *$|H\left(X|Y\right)-H\left(X^{\prime }|Y^{\prime }\right)|\leq D\left(X,X^{\prime }\right)+2D\left(Y,Y^{\prime }\right)$.*
*(iv)*  *$|I\left(X;Y\right)-I\left(X^{\prime };Y^{\prime }\right)|\leq 2\left(D\left(X,X^{\prime }\right)+D\left(Y,Y^{\prime }\right)\right)$.*

**Proof.** 

(i) By the chain rule: H(X)+H(Y|X)=H(X,Y)=H(Y)+H(X|Y), hence |H(X)−H(Y)|=|H(X|Y)−H(Y|X)|≤H(X|Y)+H(Y|X)=D(X,Y).
(ii) Applying the inequality (i) to the variables (X,Y) and $\left(X^{\prime },Y^{\prime }\right)$, we obtain $|H\left(X,Y\right)-H\left(X^{\prime },Y^{\prime }\right)|\leq D\left(\left(X,Y\right),\left(X^{\prime },Y^{\prime }\right)\right)$. From the continuity of joint information (Proposition 20), one can further bound $D\left(\left(X,Y\right),\left(X^{\prime },Y^{\prime }\right)\right)\leq D\left(X,X^{\prime }\right)+D\left(Y,Y^{\prime }\right)$.
(iii) By the chain rule, $|H\left(X|Y\right)-H\left(X^{\prime }|Y^{\prime }\right)|=|H\left(X,Y\right)-H\left(Y\right)-\left(H\left(X^{\prime },Y^{\prime }\right)-H\left(Y^{\prime }\right)\right)|\leq |H\left(X,Y\right)-H\left(X^{\prime },Y^{\prime }\right)|+|H\left(Y^{\prime }\right)-H\left(Y\right)|$. The conclusion now follows from (i) and (ii).
(iv) By the chain rule, $|I\left(X;Y\right)-I\left(X^{\prime };Y^{\prime }\right)\left|=\right|H\left(X\right)-H\left(X^{\prime }\right)+H\left(Y\right)-H\left(Y^{\prime }\right)+H\left(X^{\prime },Y^{\prime }\right)$$-H\left(X,Y\right)|\leq |H\left(X\right)-H\left(X^{\prime }\right)|+|H\left(Y\right)-H\left(Y^{\prime }\right)|+|H\left(X^{\prime },Y^{\prime }\right)-H\left(X,Y\right)|$. The conclusion follows from bounding each of the three terms in the sum using (i) and (ii). □

In the remainder of this paper, we only consider quantities that are *continuous* with respect to the entropic metrics (Shannon and Rajski distance). As a result, the discontinuity of the ∧ operator will not hinder our derivations in the sequel.

## 4. Geometric Properties of the Information Lattice

### 4.1. Alignments of Random Variables

**Definition 6**  (Alignment)**.** *Let δ be any distance on the information lattice. The random variables X, Y, and Z are said to be* aligned *with respect to δ if the triangular inequality is met with equality:*

(19) δ(X,Y)+δ(Y,Z)=δ(X,Z).

**Proposition 22**  (Alignment with respect to the Shannon distance *D*)**.** *The random variables X, Y, and Z are aligned with respect to D if and only if X−Y−Z is a Markov chain and Y≤X∨Z.*

**Figure 4:**
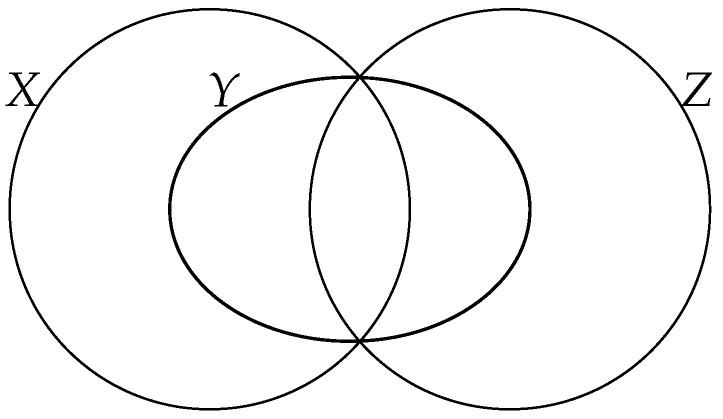
Venn diagram illustrating the alignment condition for the Shannon distance.

This alignment condition is illustrated in Figure 4.

**Proof.**  From the proof of the triangular inequality for *D* (Proposition 15), the equality holds iff equality holds in both inequalities H(X|Z)≤H(X,Y|Z)=H(X|Y,Z)+H(Y|Z)≤H(X|Y)+H(Y|Z) and those inequalities obtained by permuting the roles of *X* and *Z*. Since H(X,Y|Z)−H(X|Z)=H(Y|X,Z) and H(X|Y)−H(X|Y,Z)=I(X;Z|Y), the equality holds iff H(Y|X,Z)=0 and I(X;Z|Y)=0, both conditions being symmetric in (X,Z). Now, H(Y|X,Z)=0 means that *Y* is a function of (X,Z), i.e., Y≤X∨Z. Also, I(X;Z|Y)=0 means that *X* and *Z* are conditionally independent given *Y*, which characterizes the fact that X−Y−Z forms a Markov chain. □

**Proposition 23**  (Alignment with respect to Rajski’s distance *d*). *The random variables X, Y, and Z are aligned with respect to d if and only if either X=Y, Y=Z, or Y=X∨Z.*

**Figure 5:**
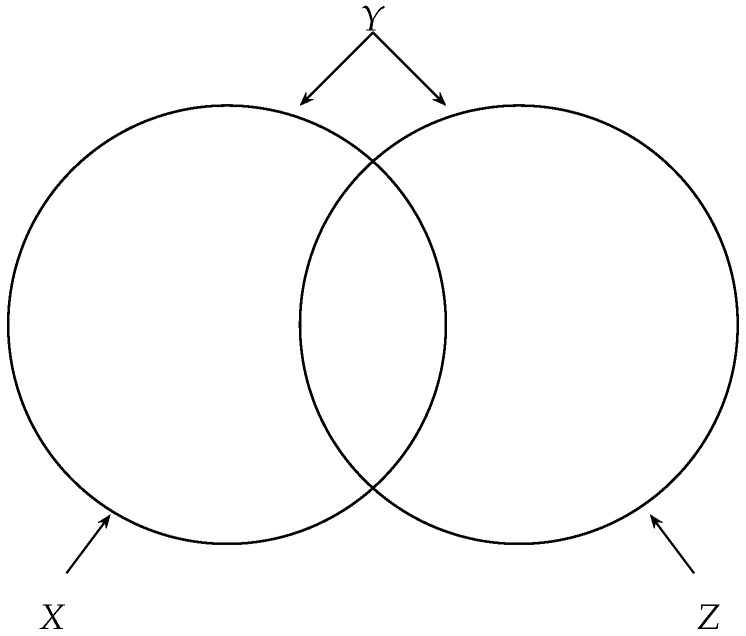
Venn diagram illustrating the alignment condition for the Rajski distance.

This alignment condition Y=X∨Z is illustrated in Figure 5.

**Proof.**  Alignment trivially holds when X=Y or Y=Z. More generally, from the proof of the triangular inequality for *d* (Proposition 16), alignment holds iff the equality holds in all inequalities (9), (10), and (12) and those inequalities obtained by permuting the roles of *X* and *Z*.

Now, a close inspection of (9) shows that it achieves equality iff H(X|Y)=0 or H(Z|Y)=0, that is X≤Y or Z≤Y. This condition is written as X∨Z≤Y and is already symmetric in X,Z.

Similarly, (10) achieves equality iff H(Y|Z)=0 or H(X|Y)=0, that is Y≤Z or X≤Y. Permuting the roles of X,Z, we also have the condition Y≥Z or X≥Y. Thus, leaving aside the trivial solutions X=Y or Y=Z, one necessarily has either X≤Y or Z≤Y, which is the same as the equality condition in (9), or the opposite inequalities, X≥Y and Z≥Y. In this latter case, combining with the equality condition in (9), we again end up with the trivial solutions X=Y or Y=Z.

Thus, leaving aside the trivial solutions X=Y or Y=Z, both conditions are written as X∨Z≤Y, which is symmetric in (X,Z). Finally, (12) achieves equality iff H(X|Y)+H(Y|Z)=H(X|Z) and the corresponding equality obtained by permuting the roles of *X* and *Z*. This means that *X*, *Y*, and *Z* are aligned with respect to *D*, that is X−Y−Z is a Markov chain and Y≤X∨Z. Overall, Y=X∨Z, which already implies that *X* and *Z* are conditionally independent given Y=(X,Z), i.e., X−Y−Z is a Markov chain. □

**Remark 12.**  *Note that if X, Y, and Z are aligned in the sense of Rajski’s distance, then they are also aligned in the sense of Shannon’s entropic distance since Y=(X,Z) implies that X−Y−Z is a Markov chain. Thus, the alignment condition is stronger in the case of the Rajski distance.*

**Remark 13.**  *The alignment condition with respect to the Rajski distance is simpler and expressed by using only the operators of the information lattice, whereas that with respect to the Shannon distance requires the additional notion of the Markov chain. Therefore, in the sequel, we develop some geometrical aspects of the information lattice based essentially on the Rajski distance.*

### 4.2. Convex Sets of Random Variables in the Information Lattice

**Definition 7**  (Convexity)**.** *Given two random variables X and Y, we define the* segment *[X,Y] of endpoints X and Y as the set of all random variables Z such that X, Z, and Y are aligned with respect to the Rajski distance, i.e., such that d(X,Z)+d(Z,Y)=d(X,Y).*

*We say that a set C of points (random variables) in the information lattice is* convex *if, for all points X,Y∈C, the segment [X,Y]⊆C. If S is any set of points of the information lattice, its* convex envelope *is the smallest convex set containing S.*

By its very definition, the convex envelope of the two-element set {X,Y} is the segment [X,Y]. We have the following simple characterization.

**Proposition 24**  (Segment characterization)**.** *For any two elements X and Y of the information lattice, the segment [X,Y] is the three-element set $\left[X,Y\right]=\left\{X,(X,Y),Y\right\}$, with the respective distances to the endpoints given by $d\left(X,(X,Y)\right)=\frac{H(Y|X)}{H(X,Y)}$ and $d\left(Y,(X,Y)\right)=\frac{H(X|Y)}{H(X,Y)}$.*

**Proof.**  *X* and *Y* do belong to the segment [X,Y] since d(X,X)+d(X,Y)=d(X,Y) and d(X,Y)+d(Y,Y)=d(X,Y). Moreover, if Z∈[X,Y], then *X*, *Z*, and *Y* are aligned with respect to the Rajski distance so that, necessarily, Z=(X,Y). One calculates $d\left(X,(X,Y)\right)=\frac{H(X|X,Y)+H(X,Y|X)}{H(X,Y)}=\frac{H(Y|X)}{H(X,Y)}$ and similarly for $d\left(Y,(X,Y)\right)$ by permuting the roles of *X* and *Y*. □

**Figure 6:**
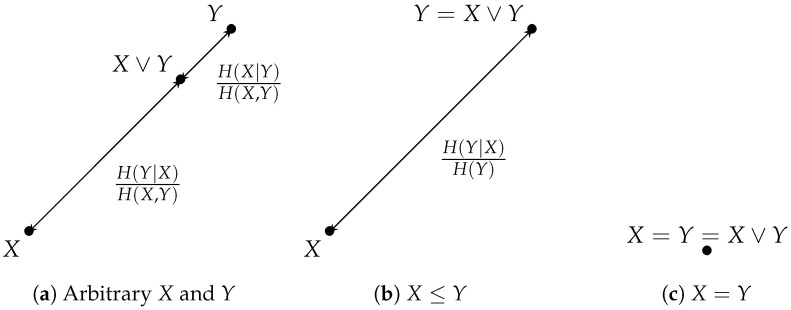
Visualization of the segment [X,Y] for three possible cases.

**Remark 14.**  *By the above Proposition, segments in the information lattice are intrinsically* discrete *objects. In the case where X≤Y or Y≤X, then the segment [X,Y] contains only two distinct points, X and Y. Obviously, if X=Y, then [X,Y] is a singleton. This gives three possible cases as illustrated in Figure 6.*

**Remark 15.**  *As a result of this characterization, four or more distinct points cannot be aligned with respect to the Rajski distance, because a segment cannot contain more than three distinct points.*

**Proposition 25.**  *C is convex iff it is closed under the ∨ operator.*

**Proof.**  C is convex iff, for all X,Y∈C, [X,Y]⊆C, that is *X*, *Y*, and (X,Y)=X∨Y∈C. □

Beyond the case of a two-element set, we now characterize the convex envelope of any *n*-element set in the information lattice, that is the convex envelope of *n* random variables $X_{1}$, $X_{2}$,…, $X_{n}$. We adopt the following usual convention. For any *n*-tuple of indices $I=(i_{1},i_{2},\ldots ,i_{n})$, the random vector $\left(X_{i_{1}},X_{i_{2}},\ldots ,X_{i_{n}}\right)=X_{i_{1}}\vee X_{i_{2}}\vee \cdots \vee X_{i_{n}}$ is denoted by $X_{I}$. Again, by convention, for the empty set, $X_{\emptyset }=0$, so that one always has $X_{I\cup J}=X_{I}\vee X_{J}$ for any two finite sets of indices *I* and *J*.

**Proposition 26.**  *Let I be a finite index set and ${\left(X_{i}\right)}_{i\in I}$ be random variables. The convex envelope of ${\left(X_{i}\right)}_{i\in I}$ is $\left\{\vee_{j\in J}X_{j}|\emptyset \neq J\subseteq I\right\}=\left\{X_{J}|\emptyset \neq J\subseteq I\right\}$, that is the set of all sub-tuples of the $X_{i}$.*

**Proof.**  With every $X_{i}$ (i∈I), the convex envelope in question should be closed by the ∨ operator, hence contain any tuple $\vee_{j\in J}X_{j}$ for any nonempty J⊆I. Now, $C=\{\vee_{j\in J}X_{j}=X_{J}|\emptyset \neq J\subseteq I\}$ contains all $X_{i}$ for i∈I and is already convex. Indeed, for all $X_{J}\in C$ and $X_{K}\in C$, $X_{J}\vee X_{K}=X_{J\cup K}\in C$. □

**Remark 16.**  *Given a finite set I of an index of cardinality |I|=n, the convex envelope of ${\left(X_{i}\right)}_{i\in I}$ contains at most $2^{n}-1$ distinct elements, since there are $2^{n}-1$ nonempty subsets of I. The number $2^{n}-1$ is only an upper bound since it might happen that two different subsets J and K of I are such that $X_{J}=X_{K}$.*

**Figure 7:**
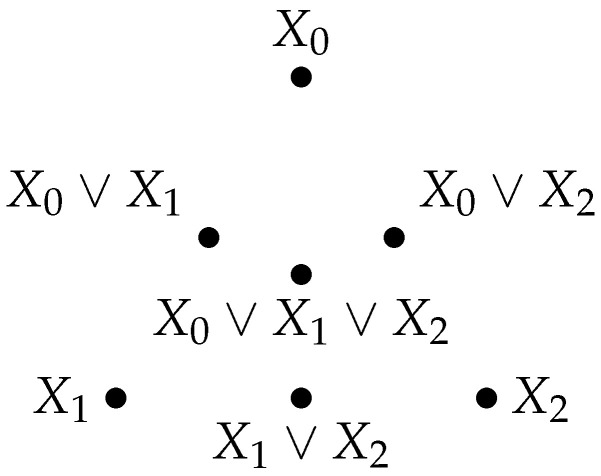
Seven-element convex envelope of three random variables $X_{0}$, $X_{1}$, and $X_{2}$. These three random variables are represented as vertices of an (equilateral) triangle. The other points in the convex envelope are obtained as intersections of medians and edges, and the common information $X_{0}\vee X_{1}\vee X_{2}$ is the center of gravity (intersection of the three medians). Similarly, the 15-element convex envelope of four distinct points can be visualized as a tetrahedron, etc.

An example of the convex envelope of a family of three random variables is shown in Figure 7.

It is also interesting to note that any sublattice of the information lattice does have some convexity properties:

**Proposition 27.**  *Any sublattice of the information lattice (including the information lattice itself) is* convex *in the sense of Definition 7. If a sublattice contains a subset of points ${\left(X_{i}\right)}_{i}$, it also contains every point in the convex envelope of ${\left(X_{i}\right)}_{i\in I}$.*

**Proof.**  With every two points X,Y, the sublattice should contain their maximum X∨Y, hence the whole segment [X,Y]. It is, therefore, convex. Now, every convex set contains the convex envelope of any of its subsets. □

### 4.3. The Lattice Generated by a Random Variable

In the sequel, we are interested in all possible deterministic functions of a given random variable *X*. In fact, their set constitutes a sublattice of the information lattice:

**Proposition 28**  (Sublattice generated by a random variable)**.** *Let X be any random variable in the information lattice. The set of all random variables ≤X is a sublattice, which we call* lattice generated by *X, denoted 〈X〉. It is a bounded lattice with maximum (total information) X and minimum 0.*

**Proof.**  Let Y≤X and Z≤X. There exists deterministic functions *f* and *g* such that Y=f(X) a.s. and Z=g(X) a.s. Clearly, Y∧Z≤Y≤X and Y∨Z=(Y,Z)=(f(X),g(X))≤X. Therefore, the set of random variables ≤X forms a sublattice. Clearly, *X* is maximum, and 0 (deterministic random variable seen as a constant function of *X*) is minimum. □

**Remark 17.**  *One may also define the sublattice $\langle X_{1},X_{2},\ldots ,X_{n}\rangle$ generated by several random variables $X_{1},X_{2},\ldots ,X_{n}$ simply as the sublattice generated by the variable $X_{1}\vee X_{2}\vee \cdots \vee X_{n}$. Therefore, it is enough to restrict ourselves to one random variable X as the lattice generator.*

**Proposition 29.**  *〈X〉 is a complemented lattice.*

**Proof.**  Let Y≤Z≤X, so that both Y,Z∈〈X〉. By Proposition 8, *Y* admits at least one complement information $\bar{Y}$ with respect to *Z* in the information lattice, such that $Y\wedge \bar{Y}=0$ and $Y\vee \bar{Y}=Z$. Now, $\bar{Y}\leq Z\leq X$; hence, the complement $\bar{Y}\in \langle X\rangle$ belongs to the sublattice generated by *X*. □

### 4.4. Properties of Rajski and Shannon Distances in the Lattice Generated by a Random Variable

We now investigate the metric properties of the sublattice 〈X〉 generated by a random variable *X*. To avoid the trivial case 〈0〉={0}, we assume that *X* is *nondeterministic*. First of all, we observe that the entropy of an element of the sublattice increases as it is closer to *X* (in terms of either Shannon’s or Rajski’s distance):

**Proposition 30.**  *For any Y∈〈X〉, one has D(X,Y)=H(X|Y)=H(X)−H(Y) and $d\left(X,Y\right)=\frac{H(X|Y)}{H(X)}=1-\frac{H(Y)}{H(X)}$. In particular, the maximum distance d(X,Y)=1 is achieved iff Y=0.*

**Proof.**  One has

(20) $$\begin{array}{cc} d(X,Y) & =\frac{D(X,Y)}{H(X,Y)}=\frac{H(X|Y)+H(Y|X)}{H(X)}\\ \; & \overset{\left(a\right)}{=}\frac{H(X|Y)}{H(X)}\overset{\left(b\right)}{=}\frac{H(X)-H(Y)}{H(X)}=1-\frac{H(Y)}{H(X)}. \end{array}$$

where (a) is because Y≤X and (b) is a consequence of the chain rule: H(X)=H(X,Y)=H(Y)+H(X|Y). □

**Remark 18.**  *In the language of data compression, $d\left(X,Y\right)=\frac{H(X)-H(Y)}{H(X)}=1-\frac{H(Y)}{H(X)}$ can be seen as the relative entropic* redundancy *of X when it is represented (“encoded”) by Y.*

**Remark 19.**  *The maximum distance case in the proposition can be stated as follows: The only random variables Y that can be obtained as functions of X (Y∈〈X〉) while being also independent of X (d(X,Y)=1) are the constant (deterministic) random variables.*

### 4.5. Triangle Properties of the Shannon Distance

At least one attempt has been made previously by Donderi [18,19] to relate the entropic distance *D* to Euclidean geometry. Referring to Shannon’s lattice of information, Donderi defined the distance between *X* and *Y* to be $\sqrt{D(X,Y)}=\sqrt{H(X|Y)+H(Y|X)}$, rather than D(X,Y), and postulated that such a distance satisfies the usual properties of a Euclidean distance, such as the trigonometric law of cosines for a triangle (see Figure 1 in [18] and Figure 2 in [19]). This, in fact, is not the case, and the geometry of the triangle has to be re-thought in a non-Euclidean way as follows.

In Euclidean geometry, *Apollonius’s theorem* allows one to calculate the length of the median of a triangle XYZ given the length of its other three sides. In the information lattice context, Y∨Z denotes the median of the segment [Y,Z] (the only possible point in the segment that is not an endpoint). Thus, Apollonius’s theorem gives a formula for the distance D(X,Y∨Z) in terms of D(X,Y), D(X,Z), and D(Y,Z). The following Proposition is the analogue of Apollonius’s theorem for the Shannon distance in the information lattice generated by *X*:

**Lemma 3**  (Apollonius’s theorem in 〈X〉)**.** *For any Y,Z∈〈X〉,*

(21) $$D\left(X,Y\vee Z\right)=\frac{D(X,Y)+D(X,Z)-D(Y,Z)}{2}.$$

*This can also be written as*

(22) D(X,Y)+D(X,Z)=D(Y,Z)+2D(X,Y∨Z)

**Figure 8:**
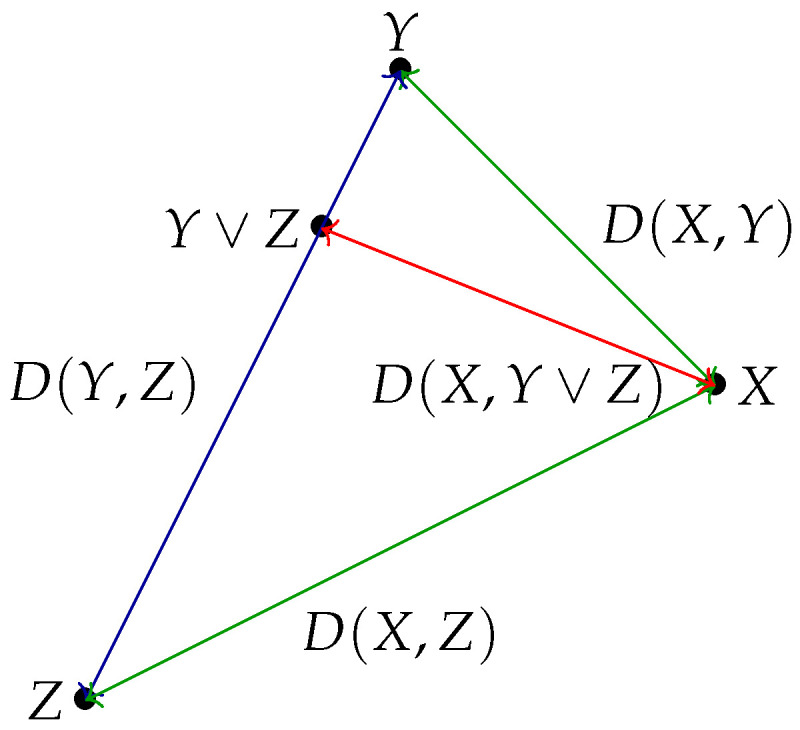
Graphical representation of Apollonius’s theorem (Lemma 3).

This is illustrated in Figure 8. Note that, when X=Y∨Z, one recovers that Y,X,Z (in this order) are aligned.

**Proof.**  From Proposition 30, D(X,Y)=H(X)−H(Y) for any Y∈〈X〉, in particular D(X,Z)=H(X)−H(Z) and D(X,Y∨Z)=H(X)−H(Y,Z) also. Therefore, D(X,Y)+D(X,Z)−2D(X,Y∨Z)=2H(Y,Z)−H(Y)−H(Z)=H(Z|Y)+H(Y|Z)=D(Y,Z). □

From Lemma 3, we derive the following,

**Lemma 4.**  *For any Y,Z∈〈X〉,*

(23) d(X,Y)+d(X,Z)≤d(X,Y∨Z)+1

*with equality if and only if Y and Z are independent.*

**Proof.**  Observe that D(Y,Z)+D(X,Y∨Z)=H(Y|Z)+H(Z|Y)+H(X|Y∨Z)≤H(Y)+H(Z|Y)+H(X|Y,Z)=H(Y,Z)+H(X|Y,Z)=H(X,Y,Z)=H(X) since Y,Z∈〈X〉, with equality iff *Y* and *Z* are independent. Now, by Lemma 3, D(X,Y)+D(X,Z)=D(Y,Z)+2D(X,Y∨Z)≤D(X,Y∨Z)+H(X). Dividing by H(X)=H(X,Y)=H(X,Z)=H(X,Y,Z) yields the announced inequality. □

In the other direction, we have the following.

**Lemma 5.**  *For any Y,Z∈〈X〉,*

(24) d(X,Y∨Z)≤d(X,Y)+d(X,Z)

*with equality if and only if X=Y=Z.*

**Proof.**  By the triangular inequality, d(X,Y∨Z)≤d(X,Y)+d(Y,Y∨Z) with equality iff Y=X∨Y∨Z=X by the alignment condition. Similarly, d(X,Y∨Z)≤d(X,Z)+d(Z,Y∨Z) with equality iff Z=X∨Y∨Z=X. Summing the two inequalities, 2d(X,Y∨Z)≤d(X,Y)+d(X,Z)+d(Y,Y∨Z)+d(Z,Y∨Z), where d(Y,Y∨Z)+d(Z,Y∨Z)=d(Y,Z)≤d(X,Y)+d(X,Z) with equality iff X=Y∨Z. Combining yields the announced inequality. □

**Remark 20.**  *In the course of the proof, we proved the following stronger inequality: for any Y,Z∈〈X〉,*

(25) $$d\left(X,Y\vee Z\right)\leq \frac{d(X,Y)+d(Y,Z)+d(Z,X)}{2}$$

*with the same equality condition X=Y=Z.*

**Remark 21.**  *By Lemmas 4 and 5, we see that, in terms of the Rajski distances to the generator X, d(X,Y∨Z) lies between d(X,Y)+d(X,Z)−1 and d(X,Y)+d(X,Z), where the lower and upper bounds differ by one and the minimum value is achieved in the case of independence. These two Lemmas are instrumental in the derivations of the next section.*

## 5. The Perfect Reconstruction Problem

### 5.1. Problem Statement

Suppose one is faced with the following reconstruction problem. We are given a (discrete) source of information *X* (e.g., a digital signal, some text document, or any type of data), which is processed using deterministic functions into several “components”:

(26) $$X_{1}=f_{1}\left(X\right),\;X_{2}=f_{2}\left(X\right),\;\ldots ,\;X_{n}=f_{n}\left(X\right)$$

(e.g., different filtered versions of the signal at various frequencies, translated parts of the document, or some nonlinear transformations of the data). The natural question is: Did one *loose information* when processing *X* into its *n* components $X_{1},X_{2},\ldots ,X_{n}$, or else, can we *perfectly reconstruct* the original *X* from its *n* components using some (unknown) deterministic function $X=f(X_{1},\ldots ,X_{n})$?

We emphasize that all involved functions must be *deterministic* (no noise is involved), otherwise *perfect* reconstruction (without error) would not be possible. Yet, we do not require any precise form for the reconstruction function *f*, only that such a reconstruction exists. To our knowledge, the first occurrence of such a problem (for n=2) is Exercise 6 of the textbook [20].

Stated in the information lattice language, the perfect reconstruction problem is as follows. Suppose we are given $X_{1},X_{2},\ldots ,X_{n}$ in 〈X〉, the sublattice generated by *X*. Is it true that $X\leq X_{1}\vee X_{2}\vee \cdots \vee X_{n}$? Since the sublattice is convex (Proposition 27), i.e., stable by the ∨ operator (Proposition 25), one always has, by assumption, that $X_{1}\vee X_{2}\vee \cdots \vee X_{n}\in \langle X\rangle$, i.e., $X_{1}\vee X_{2}\vee \cdots \vee X_{n}\leq X$. Therefore, in the reconstruction problem, it is equivalent to determining whether $X=X_{1}\vee X_{2}\vee \cdots \vee X_{n}$ or $X\neq X_{1}\vee X_{2}\vee \cdots \vee X_{n}$.

**Remark 22.**  *Geometrically, by Proposition 26, determining whether $X\leq X_{1}\vee X_{2}\vee \cdots \vee X_{n}$ or not is equivalent to determining whether X is in the* convex envelope *of ${\left(X_{i}\right)}_{i=1,\ldots ,n}$.*

**Figure 9:**
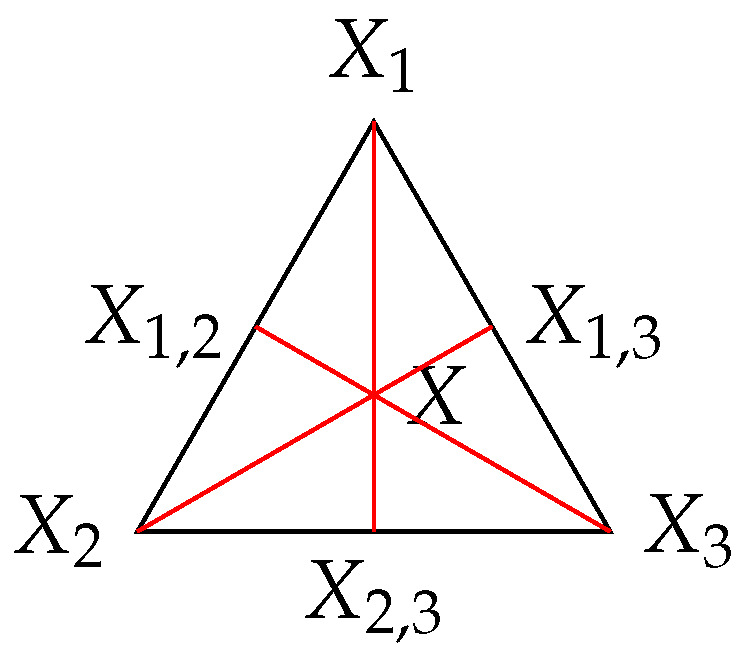
Geometric illustration of the three-component reconstruction problem.

*Thus, when n=2, perfect reconstruction is possible iff X lies in the segment $\left[X_{1},X_{2}\right]$. When n=3, perfect reconstruction is possible iff, for every distinct index i,j,k∈{1,2,3}, $X_{i}$, X, and $X_{j,k}$ are aligned with respect to the Rajski distance, as illustrated in Figure 9.*

Intuitively, the processed components $X_{i}$ should not (on the whole) be too “far away” from the original source *X* in order that perfect reconstruction be possible. In other words, at least some of the distances $d(X,X_{i})$ should not be too high. Such distances can be, in principle, evaluated when processing the source *X* into each of its components. In the following subsection, we give a simple necessary condition on the sum $d\left(X,X_{1}\right)+d\left(X,X_{2}\right)+\cdots +d\left(X,X_{n}\right)$ to allow for perfect reconstruction.

### 5.2. A Necessary Condition for Perfect Reconstruction

The main result of this paper is the following.

**Theorem 1**  (Necessary condition for perfect reconstruction)**.** *Let X be a random variable, and let $X_{1},X_{2},\ldots ,X_{n}\in \langle X\rangle$. If perfect reconstruction is possible: $X=X_{1}\vee X_{2}\vee \cdots \vee X_{n}$, then*

(27) $$\sum_{i=1}^{n}d\left(X,X_{i}\right)\leq n-1$$

*with equality iff $X_{1},X_{2},\ldots ,X_{n}$ are independent.*

**Proof.**  By repeated use of Lemma 4, each joining operation of two components in the sum—e.g., passing from $d\left(X,X_{i}\right)+d\left(X,X_{j}\right)$ to $d(X,X_{i}\vee X_{j})$—decreases this sum by at most one. Thus,

(28) $$\begin{array}{cc} \sum_{i=1}^{n}d\left(X,X_{i}\right) & \leq \sum_{i=1}^{n-2}d\left(X,X_{i}\right)+d\left(X,X_{n-1}\vee X_{n}\right)+1\\ \; & \leq \sum_{i=1}^{n-3}d\left(X,X_{i}\right)+d\left(X,X_{n-2}\vee X_{n-1}\vee X_{n}\right)+2\\ \; & \;\;\vdots \\ \; & \leq d(X,X_{1}\vee X_{2}\vee \cdots \vee X_{n})+n-1=n-1. \end{array}$$

The equality holds iff all the above n−1 inequalities are equalities. By the equality condition of Lemma 4, this means by induction that $X_{1}$ is independent of $X_{2}\vee \cdots \vee X_{n}$, where $X_{2}$ is independent of $X_{3}\vee \cdots \vee X_{n}$, and so on, until $X_{n-1}$ is independent of $X_{n}$. Overall, this is equivalent to saying that all components $X_{1},X_{2},\ldots ,X_{n}$ are mutually independent. □

**Remark 23.**  *To illustrate Theorem 1, consider a uniformly distributed two-bit random variable X (i.e., the result of two independent coin flips), and let $X_{1}$ be the result of the first coin toss and $X_{2}$ be that of the second coin toss. Clearly, reconstruction is possible since $X=(X_{1},X_{2})$. Now, a simple calculation gives $d\left(X,X_{1}\right)=\frac{H(X|X_{1})}{H(X)}=\frac{\log2}{\log4}=\frac{1}{2}$, and similarly, $d\left(X,X_{2}\right)=\frac{1}{2}$, which shows that (27) is achieved with equality: $d\left(X,X_{1}\right)+d\left(X,X_{2}\right)=2-1=1$. This is not surprising since $X_{1}$ and $X_{2}$ are independent, as can be checked directly.*

*Now, consider $X_{3}=0$ or 1 depending on whether $X_{1}=X_{2}$ or not. Clearly, X can be also reconstructed from $X_{1},X_{2},X_{3}$ since it can already be reconstructed from $X_{1},X_{2}$. Again, one computes $d\left(X,X_{3}\right)=\frac{\log2}{\log4}=\frac{1}{2}$, so in this case, the sum of the distances to X is now $d\left(X,X_{1}\right)+d\left(X,X_{2}\right)+d\left(X,X_{3}\right)=\frac{3}{2}<3-1=2$. This shows that (27) is still satisfied, but not with equality. In fact, it can easily be proven that, even though $X_{1},X_{2},X_{3}$ are* pairwise *independent, they are* not mutually *independent.*

**Remark 24.**  *In practice, Theorem 1 gives an* impossibility *condition for the perfect reconstruction of the random variable X from components $X_{1},X_{2},\ldots ,X_{n}$. Indeed, if the latter are such that*

(29) $$\sum_{i=1}^{n}d\left(X,X_{i}\right)>n-1$$

*then perfect reconstruction is* impossible*, however complex the reconstruction function f could have been. In other words, $X<X_{1}\vee X_{2}\vee \cdots \vee X_{n}$, and information was lost by processing.*

*That perfect reconstruction is impossible does not mean that it would never be possible to deduce one particular value of X from some particular values of $X_{1},X_{2},\ldots ,X_{n}$. This means that such a deduction is not possible in general, for every possible value taken by $X_{1},X_{2},\ldots ,X_{n}$. In other words, there is at least one set of values $X_{1}=x_{1}$, $X_{2}=x_{2}$, …, $X_{n}=x_{n}$ for which X cannot be reconstructed unambiguously.*

**Remark 25.**  *Another look at Theorem 1 can be made using the dependency coefficient ρ=1−d in place of the Rajski distance. Then, the impossibility condition (29) is simply written as*

(30) $$\sum_{i=1}^{n}\rho \left(X,X_{i}\right)<1.$$

*In other words, perfect reconstruction can only occur if the components are (as a whole) sufficiently dependent on the original X. Otherwise, (30) precludes perfect reconstruction.*

**Remark 26.**  *Since the Rajski distance is always upper bounded by one, if the impossibility condition (29) is met, then the actual value of the sum $\sum_{i=1}^{n}d\left(X,X_{i}\right)$ necessarily lies in the interval (n−1,n].*

*In the worst situation $\sum_{i=1}^{n}d\left(X,X_{i}\right)=n$, all terms should equal one: $d(X,X_{i})=1$. This means that all components are independent of X. By Proposition 30, the components $X_{i}=0$ are all constants: in this case, all information is lost.*

**Remark 27.**  *By Theorem 1, for perfect reconstruction to be possible, the components $X_{i}$ should be (at least slightly) tightened around X in the sense that (27) is satisfied. The example of Remark 23 shows that, under this condition (even when the inequality is strict), it may be actually possible to reconstruct X. However, proximity may not be enough: the necessary condition of Theorem 1 is* not *sufficient in general.*

*To see this, consider X uniformly distributed in the integer interval {0,1,…,11}, and define $X_{1}=k$ if X=2k or 2k+1 and $X_{2}=\ell$ if X=3ℓ, 3ℓ+1, or 3ℓ+2. In other words $X_{1}$ is the integer division of X by 2, and $X_{2}$ is the integer division of X by 3. One easily computes*

(31) $$d\left(X,X_{1}\right)+d\left(X,X_{2}\right)=\frac{H\left(X|X_{1}\right)+H\left(X|X_{2}\right)}{H(X)}=\frac{\log2+\log3}{\log12}=\frac{\log6}{\log12}<1.$$

*While the necessary condition (27) of Theorem 1 is met, the value of X cannot be unambiguously determined from those of $X_{1}$ and $X_{2}$. For example, $X_{1}=X_{2}=0$ leaves two possibilities: X=0 or 1. Therefore, perfect reconstruction is not possible.*

*Another way to see this is to observe that perfect reconstruction is equivalent to saying that $X_{1},X,X_{2}$ are aligned, which in terms of the Shannon distance would be written as $D\left(X_{1},X_{2}\right)=D\left(X,X_{1}\right)+D\left(X,X_{2}\right)$. But, while $D\left(X,X_{1}\right)+D\left(X,X_{2}\right)=\log6$, one has*

(32) $$D\left(X_{1},X_{2}\right)=H\left(X_{1}|X_{2}\right)+H\left(X_{2}|X_{1}\right)=\left(\frac{1}{3}\log3+\frac{2}{3}\log\frac{3}{2}\right)+\frac{2}{6}\log2=\log3-\frac{\log2}{3}$$

*which is clearly less than log6. Therefore, perfect reconstruction is impossible in our example, because $X_{1}$ and $X_{2}$ are too close together, i.e., there is too much redundant information between them.*

*A slight modification of the above example where X takes values in {0,1,…,12m−1} for arbitrarily large m shows that the sum $d\left(X,X_{1}\right)+d\left(X,X_{2}\right)=\frac{\log6}{\log(12m)}$ can actually be as small as desired, while perfect reconstruction is still impossible. Therefore, there can be no condition of the form $\sum_{i=1}^{n}d\left(X,X_{i}\right)<c$ (or any condition based only on the value of this sum) to ensure perfect reconstruction. Such a sufficient condition cannot be established without assuming some other property of the components $X_{i}$, as seen in the next subsection.*

### 5.3. A Sufficient Condition for Perfect Reconstruction

For independent components $X_{1},X_{2},\ldots ,X_{n}$ (with no redundant information between them), the necessary condition of Theorem 1 becomes also a sufficient condition:

**Theorem 2**  (Sufficient condition for perfect reconstruction). *Let X be a random variable, and let $X_{1},X_{2},\ldots ,X_{n}\in \langle X\rangle$ be* independent *. If the inequality (27) holds, then it necessarily holds with equality:*

(33) $$\sum_{i=1}^{n}d\left(X,X_{i}\right)=n-1$$

*and perfect reconstruction is possible: $X=X_{1}\vee X_{2}\vee \cdots \vee X_{n}$.*

**Proof.**  A closer look at the proof of Theorem 1 shows that we have established (without the perfect reconstruction assumption) the general inequality:

(34) $$\sum_{i=1}^{n}d\left(X,X_{i}\right)\leq d\left(X,X_{1}\vee X_{2}\vee \cdots \vee X_{n}\right)+n-1$$

which holds with equality iff $X_{1},X_{2},\ldots ,X_{n}$ are independent. Therefore, by the independence assumption, (27) is written as $\sum_{i=1}^{n}d\left(X,X_{i}\right)=d\left(X,X_{1}\vee X_{2}\vee \cdots \vee X_{n}\right)+n-1\leq n-1$. Since the distance is nonnegative, this necessarily implies that the inequality holds with equality and that $d(X,X_{1}\vee X_{2}\vee \cdots \vee X_{n})=0$, that is $X=X_{1}\vee X_{2}\vee \cdots \vee X_{n}$. □

**Remark 28.**  *Following Remark 26, we see that, for independent $X_{1},X_{2},\ldots ,X_{n}$, the sum of the distances to X: $\sum_{i=1}^{n}d\left(X,X_{i}\right)$ can only take values in the interval [n−1,n], with two possibilities:*

- *Either $\sum_{i=1}^{n}d\left(X,X_{i}\right)=n-1$, and perfect reconstruction is possible;*
- *Or $\sum_{i=1}^{n}d\left(X,X_{i}\right)>n-1$, and perfect reconstruction is impossible.*

*In other words, independent components cannot be arbitrarily tightly packed around X.*

*Following Remark 25, in terms of dependency coefficients, for independent $X_{1},X_{2},\ldots ,X_{n}$:*

- *Either $\sum_{i=1}^{n}\rho \left(X,X_{i}\right)=1$, and perfect reconstruction is possible;*
- *Or $\sum_{i=1}^{n}\rho \left(X,X_{i}\right)<1$, and perfect reconstruction is impossible.*

**Remark 29.**  *Following Remark 22 and Figure 9 in the case of three* independent *components $X_{1},X_{2},X_{3}$, one should have $d\left(X,X_{1}\right)+d\left(X,X_{2}\right)+d\left(X,X_{3}\right)=2$ for perfect reconstruction to hold. Incidentally, the graphical Euclidean illustration of Figure 9 is faithful in this case, since, for an equilateral triangle $X_{1}X_{2}X_{3}$ with sides of length one, the sum of the Euclidean distances equals $d\left(X,X_{1}\right)+d\left(X,X_{2}\right)+d\left(X,X_{3}\right)=\frac{2}{3}+\frac{2}{3}+\frac{2}{3}=2$.*

**Remark 30.**  *By Proposition 30, $d\left(X,X_{i}\right)=1-\frac{H(X_{i})}{H(X)}$, so that Theorems 1 and 2 can be rewritten using the standard assertions that $H\left(X\right)\leq \sum H\left(X_{i}\right)$ with equality when $X_{i}$ are mutually independent. This, of course, does not require all the machinery developed earlier. We feel, however, that our geometric vision is still valuable because of its conceptual and pedagogical interest and also as a starting point for a “perfect reconstruction theory”, which, of course, needs to be improved and further investigated along these lines.*

### 5.4. Approximate Reconstruction

Suppose we encode the information source *X* by *n* components $X_{1},X_{2},\ldots ,X_{n}$, but do not particularly insist that *perfect* reconstruction is possible. Rather, we assume that the encoding removes a *fraction of redundancy* in *X* equal to

(35) $$d(X,X_{1}\vee X_{2}\vee \cdots \vee X_{n})=\delta$$

(see Remark 18). Since the case δ=0 corresponds to the previous case of perfect reconstruction ($X=X_{1}\vee X_{2}\vee \cdots \vee X_{n}$), we assume that δ>0 in the sequel. Thus, in what follows, the reconstruction of *X* can only be approximate (up to a certain distance tolerance δ). We then have the following.

**Theorem 3**  (Approximate reconstruction)**.** *Let X be a random variable, and let $X_{1},X_{2},\ldots ,X_{n}\in \langle X\rangle$ such that (35) holds with redundancy =δ>0. Then,*

(36) $$\delta <\sum_{i=1}^{n}d\left(X,X_{i}\right)\leq n-1+\delta .$$

*with equality in the second inequality iff the components $X_{1},X_{2},\ldots ,X_{n}$ are independent.*

**Proof.**  The rightmost inequality in (36) is just (34) (with the announced case of equality), which was established by repeated application of Lemma 4. Similarly, the repeated application of Lemma 5 gives

(37) $$d\left(X,X_{1}\vee X_{2}\vee \cdots \vee X_{n}\right)\leq \sum_{i=1}^{n}d\left(X,X_{i}\right)$$

with equality iff all $X_{i}=X$ (i=1,…,n). But, such an equality condition would yield δ=d(X,X)=0, contrary to the assumption δ>0. This shows that the leftmost inequality in (36) is strict. □

**Remark 31.**  *Similarly, as in the above two subsections, one can deduce from Theorem 3 that, for independent components $X_{1},X_{2},\ldots ,X_{n}$, one necessarily has $\sum_{i=1}^{n}d\left(X,X_{i}\right)=n-1+\delta$ and that, in general, approximate reconstruction within distance tolerance ≤δ will be impossible if $\sum_{i=1}^{n}d\left(X,X_{i}\right)>n-1+\delta$.*

## 6. Examples and Applications

In this section, we develop five examples of the applications of the theorems of Section 5.

### 6.1. Reconstruction from Sign and Absolute Value

Consider a real-valued random variable *X*, and assume that it is symmetric (*X* is identically distributed as −X) and that P(X=0)=0. Now, define $X_{1}=\left|X\right|$ (absolute value) and $X_{2}=\mathrm{sgn}\left(X\right)\in \left\{-1,1\right\}$ (sign of *X*). Clearly, if *X* follows probability distribution *p*, then $X_{1}$ has probability distribution 2p(x) for x>0. Also, $X_{2}$ is Rademacher distributed (equiprobable ±1).

One easily computes $H\left(X_{1}\right)=\sum_{x>0}2p\left(x\right)\log\frac{1}{2p(x)}=H\left(X\right)-\log2$ and $H(X_{2})=\log2$ (equiprobable ±1); hence, $d\left(X,X_{1}\right)=1-\frac{H(X_{1})}{H(X)}=\frac{\log2}{H(X)}$ and $d\left(X,X_{2}\right)=1-\frac{H(X_{2})}{H(X)}=1-\frac{\log2}{H(X)}$. Therefore, $d\left(X,X_{1}\right)+d\left(X,X_{2}\right)=1$: Inequality (27) is satisfied with equality.

Of course, in this trivial example, perfect reconstruction is possible since $X=|X|\mathrm{sgn}\left(X\right)=X_{1}X_{2}$. Then, by Theorem 1, we deduce that $X_{1}$ and $X_{2}$ are independent. This is easily checked directly since, by the symmetry assumption, $P\left(X_{1}=x_{1}|X_{2}=\pm 1\right)=P\left(X_{1}=x_{1}\right)$. Notice that, from this independence, by Theorem 2, we find anew that perfect reconstruction of *X* is possible from $X_{1}$ and $X_{2}$.

This example can be easily generalized to the case of a “symmetric” complex-valued random variable *X* with modulus $X_{1}=\left|X\right|$ and argument $X_{2}=\arg\left(X\right)$, where $X_{1}$ is independent of $X_{2}$ and $X_{2}$ is uniformly distributed over *M* possible values. Then, $H(X_{2})=\log M$, $H\left(X_{1}\right)=H\left(X\right)-\log M$ by symmetry, and similar conclusions hold.

Of course, perfect reconstruction $X=X_{1}X_{2}$ is always possible even in the case where *X* is *not* symmetric, in which case $X_{1}$ and $X_{2}$ are *not* independent, and therefore, by the alignment condition, $d\left(X,X_{1}\right)+d\left(X,X_{2}\right)=d\left(X_{1},X_{2}\right)<1$.

### 6.2. Linear Transformation over a Finite Field

Consider *X* uniformly distributed over $F_{q}^{k}$, where $F_{q}$ is the field with *q* elements. Suppose *X* is linearly transformed using some matrix G to obtain

(38) $$(X_{1},X_{2},\ldots ,X_{n})=X\cdot G$$

in row vector notation, where $G\in F_{q}^{k\times n}$ has *k* rows and *n* columns. For example, *X* may represent information symbols to be transmitted over a channel, and $\left(X_{1},X_{2},\ldots ,X_{n}\right)$ would be the associated codeword using an (n,k) linear code over $F_{q}$ with generator matrix G.

If the *i*th column of G is not the all-zero vector, then it is easily checked that, since *X* is uniformly distributed over $F_{q}^{k}$, $X_{i}$ is likewise uniformly distributed over $F_{q}$. Therefore,

(39) $$d\left(X,X_{i}\right)=1-\frac{H(X_{i})}{H(X)}=1-\frac{\log q}{\log q^{k}}=1-\frac{1}{k}.$$

When the *i*th column of G is all zero, however, $d\left(X,X_{i}\right)=d\left(X,0\right)=1$. Summing up,

(40) $$\sum_{i=1}^{n}d\left(X,X_{i}\right)=n-\frac{n^{\prime }}{k}$$

where $n^{\prime }\leq n$ is the number of non-zero columns in G.

By Theorem 1, if $n-\frac{n^{\prime }}{k}>n-1$, that is $n^{\prime }<k$, then perfect reconstruction is impossible. This is quite natural since, in this case, $\left(X_{1},\ldots ,X_{n}\right)$ entails less *q*-ary symbols than the vector *X*, so that it is impossible to reconstruct *X* from the $n^{\prime }$ actual symbols in $\left(X_{1},\ldots ,X_{n}\right)$.

In general, if G has rank $r\leq \min(k,n^{\prime })$, then, since *X* is uniformly distributed over $F_{q}^{k}$, the vector $\left(X_{1},\ldots ,X_{n}\right)$ is also uniformly distributed over a subspace of $F_{q}^{n}$ of dimension *r*. Now, as we have just seen, if the *i*th column of G is not the all-zero vector, then $X_{i}$ is uniformly distributed over $F_{q}$. Since uniformly distributed components of a discrete random vector are independent iff the vector is itself uniformly distributed, the only possibility for the components $X_{1},\ldots ,X_{n}$ to be independent as in Theorem 2 is that $\left(X_{1},\ldots ,X_{n}\right)$ is uniformly distributed over $F_{q}^{n^{\prime }}$, that is $r=n^{\prime }=k$. In this case $\sum_{i=1}^{n}d\left(X,X_{i}\right)=n-1$, and by Theorem 2, perfect reconstruction is possible. Of course, from linear algebra, we know that *X* can be reconstructed from $\left(X_{1},\ldots ,X_{n}\right)$ as soon as G has rank $r=k\leq n^{\prime }$.

Due to the power of linear algebra, this example may appear quite trivial. It would be interesting to generalize it, however, to the case where the vector $\left(X_{1},\ldots ,X_{n}\right)$ is obtained by a *nonlinear* transformation, i.e., each $X_{i}$ are Boolean functions over $F_{q}$ of the components of vector *X*, e.g., described in algebraic normal form.

### 6.3. Integer Prime Factorization

Consider an integer-valued random variable *X*, uniformly distributed over {1,2,…,m}, and let n=π(m) be the number of primes not exceeding *m*. For every such prime *p*, let $X_{p}$ be the *p*-adic valuation of *X*, that is the largest exponent of *p* such that $p^{X_{p}}$ divides *X*. We know by the fundamental theorem of arithmetic that the prime factorization of *X* always exists and is unique: $X=\prod_{p}p^{X_{p}}$; hence, *X* can be reconstructed from the $X_{p}$s.

There are $\left\lfloor \frac{m}{p^{k}}\right\rfloor$ values of *X* divisible by $p^{k}$ and, therefore, $\left\lfloor \frac{m}{p^{k}}\right\rfloor \;\;-\;\;\left\lfloor \frac{m}{p^{k+1}}\right\rfloor$ values of *X* such that $X_{p}=k$. Thus, $H\left(X|X_{p}=k\right)=\log\left(\left\lfloor \frac{m}{p^{k}}\right\rfloor \;\;-\;\;\left\lfloor \frac{m}{p^{k+1}}\right\rfloor \right)\leq \log\frac{m}{p^{k}}=\log m-k\log p$, $H\left(X|X_{p}\right)\leq \log m-E\left(X_{p}\right)\log p$, and

(41) $$\sum_{p\;\mathrm{prime}\;\leq m}d\left(X,X_{p}\right)=\sum_{p\;\mathrm{prime}\;\leq m}\frac{H(X|X_{p})}{H(X)}\leq \sum_{p\;\mathrm{prime}\;\leq m}\frac{\log m-E(X_{p})\log p}{\log m}=n-\frac{\log m!}{m\log m}.$$

In the latter equality, we used the exact value $\sum_{p}E\left(X_{p}\right)\log p=\frac{\log m!}{m}$. This can be easily checked from the reconstruction formula itself, since

(42) $$\sum_{p\;\mathrm{prime}\;\leq m}E\left(X_{p}\right)\log p=E\log\prod_{p\;\mathrm{prime}\;\leq m}p^{X_{p}}=E\log X=\frac{\log m!}{m}.$$

Since logm!≤mlogm, the above upper bound is not tight enough to prove Inequality (27) of Theorem 1. It is only satisfied asymptotically as m→+∞ since, then, $\frac{\log m!}{m\log m}\to 1$. Likewise, the independence assumption of Theorem 2 is only true asymptotically: in fact, since, for distinct primes $p_{1},\ldots ,p_{\ell }$,

(43) $$P\left(X_{p_{1}}\geq k_{1},\ldots ,X_{p_{\ell }}\geq k_{\ell }\right)=\frac{1}{m}\left\lceil \frac{m}{p_{1}^{k_{1}}\cdots p_{\ell }^{k_{\ell }}}\right\rceil \to \frac{1}{p_{1}^{k_{1}}\cdots p_{\ell }^{k_{\ell }}}$$

it follows that the $X_{p}$s converge in distribution toward *independent* geometric variables with the respective parameters $1-\frac{1}{p}$.

### 6.4. Chinese Remainder Theorem

Consider an integer-valued random variable *X*, uniformly distributed over {0,1,…,k−1}, where $k=\prod_{i=1}^{n}k_{i}$ is the product of *n* pairwise coprime factors >1, and define the following remainders modulo these factors:

(44) $$\left\{\begin{array}{cc} X_{1} & \equiv X\;\mathrm{mod}\;k_{1}\\ X_{2} & \equiv X\;\mathrm{mod}\;k_{2}\\ \; & \vdots \\ X_{n} & \equiv X\;\mathrm{mod}\;k_{n}. \end{array}\right.$$

By the well-known *Chinese remainder theorem*, this system of equations has a unique solution in {0,1,…,k−1}, i.e., perfect reconstruction of *X* is possible.

Clearly, since *X* is uniformly distributed, $X_{i}$ is likewise uniformly distributed over $\left\{0,1,\ldots k_{i}-1\right\}$ so that $H\left(X_{i}\right)=\log k_{i}$, $d\left(X,X_{i}\right)=1-\frac{H(X_{i})}{H(X)}=1-\frac{\log k_{i}}{\log k}$ and

(45) $$\sum_{i=1}^{n}d\left(X,X_{i}\right)=\sum_{i=1}^{n}\left(1-\frac{\log k_{i}}{\log k}\right)=n-\frac{\log\prod_{i=1}^{n}k_{i}}{\log k}=n-1.$$

Thus, Inequality (27) of Theorem 1 is achieved with equality, which proves that $X_{1},X_{2},\ldots ,$$X_{n}$ are independent. Had we proven directly this independence, Theorem 2 would have shown that perfect reconstruction is possible. Thus, a information theoretic proof of the Chinese remainder theorem using this method amounts to proving such an independence. But, this can be performed quite similarly as the Chinese remainder theorem is classically proven.

With our present method, however, it can be easily seen that perfect reconstruction would *not* be possible if we do not use all components $X_{1},X_{2},\ldots ,X_{n}$. Indeed, suppose without loss of generality that one tries to reconstruct *X* only from $X_{1},X_{2},\ldots ,X_{n-1}$. Then, by the above calculation,

(46) $$\sum_{i=1}^{n-1}d\left(X,X_{i}\right)=\sum_{i=1}^{n-1}\left(1-\frac{\log k_{i}}{\log k}\right)=n-1-\frac{\log\prod_{i=1}^{n-1}k_{i}}{\log k}=n-2+\frac{\log k_{n}}{\log k}>n-2$$

which shows by Theorem 1 that perfect reconstruction of *X* from less than *n* remainders is impossible.

### 6.5. Optimal Sort

In this subsection, we provide a new information theoretic proof of the following.

**Theorem 4.**  *Any pairwise-comparison-based sorting algorithm has worst-case computational complexity $\geq \log_{2}k!=\Omega \left(k\log_{2}k\right)$, where k is the cardinality of the list to be sorted.*

Recall that log refers to the logarithm taken to any base, while here, more specifically, $\log_{2}$ is the logarithm to base two.

**Proof.**  Consider a finite, totally ordered list of *k* elements. It can be seen as a permutation of the uniquely sorted elements, and sorting this list amounts to finding this permutation. Let $X=(X_{1},X_{2},\ldots ,X_{k})$ be a (uniformly chosen) random permutation on {1,2,…,k}.

For i,j∈{1,…,k} with i≠j, let $X_{i,j}$ be the binary random variable taking the value 1 if $X_{i}<X_{j}$ and 0 otherwise. Clearly, $X_{i,j}\leq X$ for any i,j.

Since there are as many permutations such that $X_{i}<X_{j}$ such that $X_{i}>X_{j}$, every $X_{i,j}$ is a Bernoulli (1/2) variable (equiprobable bit). Therefore,

(47) $$d\left(X,X_{i,j}\right)=1-\frac{\log2}{\log k!}=1-\frac{1}{\log_{2}k!}.$$

Assuming *n* pairwise comparisons are made to sort the complete list, this gives

(48) $$\sum_{i,j\;(n\;\mathrm{terms})}d\left(X,X_{i,j}\right)=n-\frac{n}{\log_{2}k!}.$$

By Theorem 1, it is necessary that this value does not exceed n−1, i.e., $n\geq \log_{2}k!$ for perfect reconstruction to hold. In other words, the wort-case complexity to achieve the complete sort for *any* possible realization of the initial unsorted list requires at least $\left\lceil \log_{2}k!\right\rceil$ pairwise comparisons. □

**Remark 32.**  *This example illustrates a method to find a lower bound on the worst-case complexity of a problem. The first step is to express the instance of the problem as a random variable X. Second, one determines which pieces of information one is allowed to extract from X and models them as “observed” random variables $X_{i}\leq X$. Third, for each i, we compute the Rajski distance $d(X,X_{i})$. Finally, we use Theorem 1 to find a lower bound on the number of “observed” variables $X_{i}$ that are required to reconstruct X. We feel that such a method is interesting because it is often harder to find a lower bound on the complexity of a problem than to find an upper bound on it.*

## 7. Conclusions and Perspectives

It is an understatement to say that the “true” information theory of 1953 was not as popular as the classical theory of 1948. John Pierce, a colleague of Shannon, wrote that, “*apparently the structure was not good enough to lead to anything of great value”* [21]. We find two possible reasons for this pessimism: the fact that the lattice is not Boolean, which does not facilitate the calculations, and the discontinuous nature of the common information with respect to the entropy metric.

However, as we have shown in this paper, this lattice structure is quite helpful to understand reconstruction problems. As shown in Section 6, the implications of the resolution of perfect reconstruction problems go beyond signal processing, since the concept of information is pervasive in all fields of mathematics and of science. Thus, we believe it is important to deepen this theory, defining *information* per se, and to further generalize the reconstruction problems. It would indeed be of great interest to find a simple sufficient condition to reconstruct a variable *X* from the (not necessarily independent) components $X_{1},X_{2},\ldots ,X_{n}$.

One may legitimately argue that most examples (except in Section 6.1) assume uniform distributions, where the entropy is just a logarithmic measure of the alphabet size, and since all considered processings are deterministic, the essence of the present reconstruction problem appears more combinatorial than probabilistic. Indeed, a desirable perspective is to go beyond perfect reconstruction of discrete quantities by considering the possibility of the noisy reconstruction of discrete and/or continuous sources of information.

In a perspective closer to computer science, we used our theorems to find a lower bound on the complexity of the comparison-based sorting problem. It would be interesting to find other problems for which a lower bound on complexity can be found using our technique, especially for decision problems that are not known to be in P.

Finally, as another practical perspective for security problems, one may assume that *X* models all the possible values that can take a secret key in a given cryptographic device and that an attacker can observe *k* random values that are deterministically obtained from *X*. Such important problems have been studied, e.g., in [22] to evaluate information leakage in the execution of deterministic programs. One may use the theorems of Section 5 to find a lower bound on *k* for the attacker to be able to reconstruct the secret.

## Figures and Tables

**Table 1:** Computation of $X\wedge (Z_{1}\vee Z_{2})$ and of $\left(X\wedge Z_{1}\right)\vee \left(X\wedge Z_{2}\right)$.

ω	0	1	2	3
*X*	0	1	0	1
$Z_{1}$	1	1	2	2
$Z_{2}$	2	1	1	2
$Z_{1}\vee Z_{2}$	(1,2)	(1,1)	(2,1)	(2,2)
$X\wedge (Z_{1}\vee Z_{2})$	0	1	0	1
$X\wedge Z_{1}$	0	0	0	0
$X\wedge Z_{2}$	0	0	0	0
$\left(X\wedge Z_{1}\right)\vee \left(X\wedge Z_{2}\right)$	(0,0)	(0,0)	(0,0)	(0,0)
